# Marked Changes in One-Carbon Metabolism on a Low-Carbohydrate High-Fat Diet: A Randomized Controlled Trial (CARBFUNC)

**DOI:** 10.1016/j.tjnut.2026.101625

**Published:** 2026-06-02

**Authors:** Marianne Bråtveit, Johnny Laupsa−Borge, Tonje Bjørnstad, Cathrine Sommersten, Adrian McCann, Gülen Arslan Lied, Ottar Nygård, Gunnar Mellgren, Jutta Dierkes, Simon Nitter Dankel

**Affiliations:** 1 Centre for Nutrition, Department of Clinical Science, University of Bergen, Bergen, Norway; 2 Hormone Laboratory, Department of Medical Biochemistry and Pharmacology, Haukeland University Hospital, Bergen, Norway; 3 Bevital AS, Bergen, Norway; 4 Kunnskapskommunen Helse Omsorg Vest, Department of Ageing, Health and Care, Bergen, Norway; 5 Centre for Nutrition, Department of Clinical Medicine, University of Bergen, Bergen, Norway; 6 Section of Gastroenterology, Department of Medicine, Haukeland University Hospital, Bergen, Norway; 7 Department of Heart Disease, Haukeland University Hospital, Bergen, Norway

**Keywords:** humans, dietary carbohydrates, saturated fat, low-carbohydrate diet, ketosis, obesity, randomized controlled trial, B-vitamers, one-carbon metabolism, metabolomics

## Abstract

**Background:**

Studying the impact of dietary carbohydrates and fat on markers of one-carbon metabolism could provide important insight into distinct metabolic adaptations and how diets affect disease risk.

**Objectives:**

This study aimed to determine changes in plasma one-carbon metabolites and B-vitamers on isocaloric diets differing in carbohydrate processing or amount.

**Methods:**

A total of 193 individuals with obesity were randomly assigned to equal-energy and protein diets either with more processed, acellular carbohydrates [acellular, high-carbohydrate low-fat (A-HCLF), comparator arm], minimally processed cellular carbohydrates [cellular high-carbohydrate low-fat diet (C-HCLF)] [both designed with 45 energy percent (E%) carbohydrates and 38 E% fat], or a low-carbohydrate, high-fat (LCHF) diet designed with 8 E% carbohydrate and ∼75 E% fat, including 30 E% saturated fat. To address secondary outcomes of the trial, we analyzed changes in plasma one-carbon metabolites and B-vitamers, and dietary B-vitamin intakes after 3, 6, 9, and 12 mo, using constrained linear mixed-effect modeling.

**Results:**

After 3 mo, the relative change score after the LCHF diet was significantly different from the A-HCLF comparator for plasma α-hydroxybutyrate (LCHF/A-HCLF: +41.3%∗/–1.56%, *P* < 0.001; ∗: significant within-group change), methylmalonic acid (MMA) (–5.69%∗/+8.64%∗, *P* < 0.001), methionine (−7.81%∗/+1.44%, *P* = 0.001), nicotinamide (−19.7%∗/+14.0%, *P* = 0.007), 1-methylnicotinamide (mNAM) (−14.1%∗/+14.1%, *P* = 0.003), pyridoxal (−2.42%/+18.9%∗), 4-pyridoxic acid (−12.4%∗/+21.4%∗, *P* < 0.001), and cysteine (−3.00%∗/+1.10%). Between-group differences for most of these metabolites remained significant after 6 and 9 mo. Comparing C-HCLF with A-HCLF, significant differences in relative change scores were found for mNAM after 3 mo (C-HCLF/A-HCLF: −11.5%/+14.1%, *P* = 0.012) and 9 mo (−14.9%∗/+18.6%, *P* = 0.001). Plasma changes in one-carbon metabolites and B-vitamers showed weak correlations with dietary B-vitamins.

**Conclusions:**

Compared with A-HCLF, the LCHF diet was followed by significantly different changes in plasma α-hydroxybutyrate, MMA, methionine, nicotinamide, mNAM, pyridoxal, 4-pyridoxic acid, and cysteine. These shifts were largely independent of vitamin consumption and may rather reflect ketoadaptive mechanisms, including enhanced fatty acid oxidation and upregulated antioxidant defense. Carbohydrate quality had less impact on the one-carbon metabolites.

This trial was registered at clinicaltrials.gov as NCT03401970 (https://clinicaltrials.gov/study/NCT03401970).

## Introduction

**FIGURE 1:**
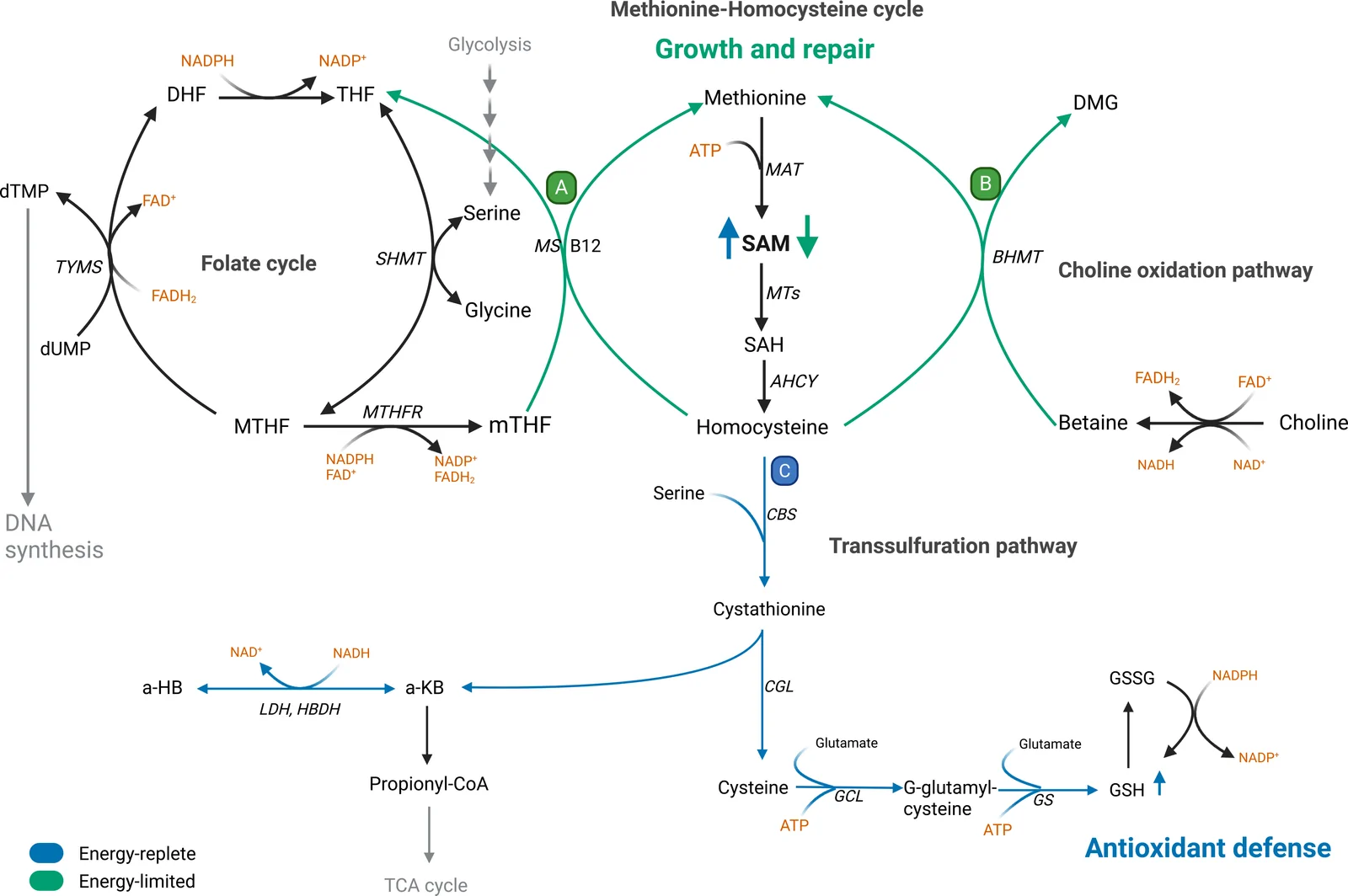
Nutrient and energy status coordinate one-carbon metabolism at the homocysteine branchpoint to balance methylation and antioxidant defense. Serine from glycolysis donates one-carbon units to the folate cycle, which supplies one-carbon groups for nucleotide synthesis and methylation reactions (DNA/histone and RNA). Methionine adenosyltransferase (MAT) uses methionine and ATP to generate S-adenosylmethionine (SAM), the universal methyl donor. After methyl transfer, SAM becomes S-adenosylhomocysteine (SAH), and then homocysteine, which either is remethylated via the (A) folate/B-12–dependent or (B) betaine-dependent remethylation pathway to regenerate methionine and sustain SAM, or (C) enters transsulfuration via cystathionine *β*-synthase (CBS) to yield cysteine for glutathione (GSH) synthesis. SAM is both a substrate and a signal: when methionine/choline/folate and ATP are sufficient, SAM rises and allosterically activates CBS, increasing transsulfuration and GSH; when methyl donors or ATP are limiting, SAM falls (being limited by both substrate supply and ATP), and cells prioritize remethylation to preserve SAM for essential methylation reactions—including DNA and histone modification, RNA processing, phosphatidylcholine synthesis for VLDL export, and polyamine production. The pentose phosphate pathway supports these processes by providing NADPH (for reductive biosynthesis and GSH recycling) and ribose-5-phosphate (for nucleotides). Together, this adaptive switch couples epigenetic, membrane-lipid, and polyamine methylation demands with redox defense according to nutrient and energy availability. Created in BioRender. Bråtveit, M. (2026) *https://BioRender.com/**kpdur61*. AHCY, adenosylhomocysteinase; α-HB, α-hydroxybutyrate; α-KB, α-ketobutyrate; BHMT, betaine-homocysteine methyltransferase; CGL, cystathionine-*γ*-lyase; DHF, dihydrofolate; DMG, dimethylglycine; dTMP, deoxythymidine monophosphate; dUMP, deoxyuridine monophosphate; GCL, glutamate–cysteine ligase; GS, glutathione synthetase; GSSG, glutathione disulfide; HBDH, hydroxybutyrate dehydrogenase; LDH, lactate dehydrogenase; MS, methionine synthase; MTHF, 5,10-methylenetetrahydrofolate; MTHFR, methylenetetrahydrofolate reductase; mTHF, 5-methyltetrahydrofolate; MTs, methyl transferases; SHMT, serine hydroxymethyltransferase; TCA, tricarboxylic acid cycle; THF, tetrahydrofolate; TYMS, thymidylate synthase.

The continuing rise in overweight and obesity promotes substantial risk of several lifestyle diseases [1]. This risk is associated with shifts in one-carbon metabolism, a pathway central for stress defense and anabolic growth, by generating S-adenosylmethionine (SAM) for essential methylation reactions (e.g., DNA and histone modification, RNA processing, phosphatidylcholine synthesis for lipid transport, and polyamine production), NADPH for reductive biosynthesis (e.g., lipids and nucleotides), and glutathione (GSH) recycling, B-vitamins—including riboflavin, niacin, vitamin B-6, folate, and vitamin B-12—serving as essential cofactors (Figure 1). Among identified risk factors, elevated plasma total homocysteine (tHcy) [2], cystathionine [3,4], choline [5,6], methylmalonic acid (MMA) [7], and dimethylglycine (DMG) [5,8] have all been associated with increased cardiovascular disease (CVD). Elevated tHcy has also been linked to cancer [9], insulin resistance [10], and type 2 diabetes [11], whereas high levels of α-hydroxybutyrate (α-HB) are associated with insulin resistance [12]. Notably, MMA has received particular attention as an established biomarker for vitamin B-12 status, a potential marker of mitochondrial dysfunction and oxidative stress, and with the proposed concentration thresholds for CVD risk [13,14].

Given the central role of one-carbon metabolism in anabolic growth, stress defense, and its links to lifestyle-related diseases such as CVD, it is important to understand how modifiable factors like diet influence this system. However, most existing studies have examined individual nutrients or food groups, almost exclusively relying on cross-sectional designs, which limits insight into the effects of overall dietary patterns. The available evidence suggest that one-carbon metabolism is sensitive to carbohydrate quality and fatty acid composition: less processed carbohydrates, low-fat dairy, and reduced processed meat intake have been associated with lower plasma tHcy and higher betaine, choline, and serine [[15], [16], [17], [18]], whereas simple sugars and other refined carbohydrates, processed meats, and fats have been associated with higher tHcy and reduced betaine levels [[15], [16], [17],^19^,^20^]. Because of the associations with food processing, another possible aspect of carbohydrate quality that might influence one-carbon metabolism is the cellularity of the food, as opposed to “acellular” foods where the cellular structures/food matrix have been degraded [21]. For fatty acid composition, increased PUFA intake was associated with higher plasma choline oxidation metabolites and lower tHcy [22], whereas statistically modeled replacement of PUFAs or MUFAs with SFAs was linked to higher plasma tHcy, MMA, and riboflavin, and lower pyridoxal-5′-phosphate (PLP) [23]. However, aside from 1 study showing that a carbohydrate-restricted diet (<30 g of carbohydrates per day) increased serum folate concentrations, upregulated folate-mediated one-carbon metabolism, and increased de novo GSH synthesis [24], there is a lack of controlled human studies examining how the overall dietary macronutrient pattern, rather than single nutrients and food groups, affects one-carbon metabolite markers and pathways, leaving a critical knowledge gap.

A particularly interesting entry point for studying fundamental dietary influences on one-carbon metabolism is a low-carbohydrate high-fat (LCHF) diet, which induces a profound shift in substrate utilization from glucose to fatty acids and ketones, and which is suspected to increase CVD risk due to high SFA intake [25,26]. This energetic switch involves one-carbon pathways, which are sensitive to energy status, NADH/NAD+ ratio, and SAM availability. In addition, different macronutrient profiles also influence B-vitamin intake and status; for example, it has been suggested that ketogenic LCHF diets may be nutritionally inadequate in water-soluble B-vitamins due to reduced consumption of fruits, vegetables, and whole grains [27,28].

In the present study, we analyzed secondary outcome metabolite data from a randomized controlled trial in males and females with obesity assigned to 1 of 3 diets: an acellular, high-carbohydrate low-fat (A-HCLF) diet, a cellular (minimally processed carbohydrates, C-HCLF) diet, or an LCHF diet, all low in added sugar. We show that the SFA-rich LCHF diet affected plasma one-carbon metabolites and B-vitamers compared with the comparator A-HCLF diet.

## Methods

### Study design and participants

In this paper, we report secondary outcome measures from the CARBFUNC trial [29], which was a 3-armed randomized controlled trial lasting for 2 y that investigated the effects of 3 isocaloric eating patterns differing in carbohydrate amount and quality in 192 individuals with obesity, with visceral fat volume as the primary outcome (clinicaltrials.gov identifier: NCT03401970). The A-HCLF diet, a relatively healthy version of the habitual diet of the study population, was used as the comparator to assess the effects of carbohydrate quality (cellular compared with acellular) and carbohydrate quantity (LCHF compared with acellular) on study outcomes, with analyses focused on these prespecified contrasts. The secondary outcomes in the present study were between-group differences in plasma one-carbon metabolites and B-vitamers, and how changes in dietary B-vitamin intakes correlated with changes in plasma B-vitamers.

The study was conducted from January 2018 to March 2021 at the Research Unit for Health Surveys, University of Bergen, Norway. Inclusion criteria were participants aged 20 to 55 y with obesity (BMI ≥30 kg/m^2^ and/or waist circumference ≥88 cm for females or ≥102 cm for males), and stable body weight (<5% change in the past 2 mo). Exclusion criteria were food allergies or intolerances affecting adherence to the diet, smoking, use of diabetes medications and/or statins, and antibiotic use or surgery treatment within the past 2 mo. Other exclusion criteria were alcohol intake >2 units/d, chronic inflammatory bowel disease, cholecystectomy, or other known diseases. In addition, postmenopausal as well as pregnant or lactating females were excluded.

The present study was conducted according to the Declaration of Helsinki and approved by the Regional Committee for Medical and Health Research Ethics of Western Norway (2017/621/REC West). All participants provided written informed consent before screening and consequent enrollment.

### Data collection

The CARBFUNC study lasted for 2 y, during which participants attended study visits at baseline, 3, 6, 9, 12, and 24 mo. In this paper, we present data from baseline to 12 mo, when participants adhered better to the diet plans, supported by structured consultations at a higher frequency (at least every 6 wk, compared with every 3 to 6 mo during the last year, as per design). Anthropometry measurements and blood sampling were performed at each study visit, after a fast for ≥12 h and avoiding strenuous exercise for the last 48 h and alcohol for the last 24 h. Additional details regarding the collection of data can be found in the original publication [29].

### Study intervention

Participants were randomly assigned to 1 of 3 isocaloric eating patterns (2000 kcal/d for females, 2500 kcal/d for males): an A-HCLF diet (comparator arm), a C-HCLF diet, or an LCHF diet. These diets were designed to differ in carbohydrate amount and processing, where carbohydrate processing was defined according to the degree of cellularity, i.e., intact cellular structures [29]. The A-HCLF diet was based on acellular carbohydrate sources, which included bakery and other mostly flour-based products (but with >50% of the flour as whole grain to limit confounding by differences in fiber content), whereas the C-HCLF diet emphasized minimally processed carbohydrates with intact cellular structures such as nonflour whole grains (e.g., whole kernels, rolled oats, and rice), fruits, and potatoes. Both HCLF diets were otherwise planned to have the same macronutrient profiles, consisting of 45 energy percent (E%) carbohydrates, 38 E% fat, and 17 E% protein, whereas the LCHF diet was planned to provide 8 E% carbohydrates, 75 E% fat, and 17 E% protein.

Within these macronutrient or cellularity profiles, all participants were advised to follow an otherwise healthy eating pattern by consuming a minimum of 500 g of fruits and/or vegetables per day and having a low intake of added sugar (≤1 E% for C-HCLF and LCHF, and ≤5 E% for A-HCLF). Participants were also instructed to avoid beverages and other products with artificial sweeteners. To facilitate adherence to the diets, a booklet with >175 diet-specific recipes was provided to the participants, where each recipe corresponded exactly to the planned overall macronutrient profile of the given diet. Additional information concerning the study diets and procedures to make the recipes can be found elsewhere [29].

### Dietary recordings and adherence

During the intervention, participants were asked to record their dietary intake by entering the IDs of the chosen recipes, or each ingredient manually, following weighing with kitchen scales that were provided. Repeated recordings were planned for 3 consecutive days every second week (2 weekdays and 1 weekend day) using an online dietary recording system (www.diett.no; operated by Dietika AS). The procedures used for calculating nutrient intakes throughout the intervention have been reported previously [30]. Self-reported adherence to the study diets was also measured by asking the participants to fill in a questionnaire at every 3-mo study visit, rating their dietary adherence from “no adherence” (0%) to complete adherence (100%) with increments of 20%.

### Anthropometry

Body weight and height were measured at each study visit. Weight was measured by a class III–approved calibrated scale (Seca 877, Seca) and height by a portable stadiometer (Seca 217, Seca). Measurements of fat mass were monitored using bioelectrical impedance (Seca mBCA 514).

### Biochemical analyses

The fasting blood samples taken at every study visit were stored at −80°C until analyzed for the study-specific analyses reported in this study, namely one-carbon metabolites and associated B-vitamers. These analyses were carried out by Bevital AS (www.bevital.no) using gas chromatography–tandem mass spectrometry (GC-MS/MS), liquid chromatography–tandem mass spectrometry (LC-MS/MS), or microbiological assays. Specifically, plasma tHcy, MMA, cysteine, methionine, serine, glycine, sarcosine, cystathionine, and α-HB were measured using GC-MS/MS [31], whereas plasma choline, betaine, DMG, PLP, pyridoxal (PL), 4-pyridoxic acid (PA), riboflavin, FMN, nicotinamide (NAM), 1-methylnicotinamide (mNAM), and folate were measured using LC-MS/MS [32]. Plasma cobalamin was measured using a microbiological assay [33].

### Randomization

Study personnel without direct involvement in the study implementation randomly allocated the participants to 1 of the 3 study diets. The randomization was performed using block randomization with block sizes ranging from 6 to 9, and stratified by sex, using the R package *blockrand* (version 1.5, RStudio, Inc.). Because of the nature of the study, both participants and study personnel were aware of their assignment to the dietary groups. The personnel at Bevital AS who analyzed plasma metabolite samples were blinded to the participants’ group assignments.

### Statistical analyses

The statistical analyses were performed using R v.4.1.2 (www.r-project.org). Data transformation and exploration were conducted using the *tidyverse* packages (https://tidyverse.tidyverse.org). The figures were made in R v.4.3.1 using the *ggplot2* package v.3.4.4. Secondary outcomes in this exploratory study were between-group differences in relative changes from baseline to follow-up (3, 6, 9, and 12 mo) of plasma one-carbon metabolites and B-vitamers, estimated in all participants (pooled analysis). We also report between-group differences in dietary B-vitamin intakes. All randomly assigned participants (*n* = 192) were included in the intention-to-treat (ITT) analysis.

Continuous study outcomes were analyzed by baseline-adjusted constrained linear mixed-effects models (cLMMs), with “subjects” as the random factor to account for repeated measurements, by using the *lme* function in the *nlme* package v3.1-150. We followed a design-driven approach and defined for the pooled analysis a fixed-effects structure including “time,” “sex,” “age,” “rac” (randomized as a couple), and “group × time,” chose a random effects structure with subject-specific intercepts and slopes for “time,” and used the first-order autoregressive correlation matrix as the covariance structure conditional on the residuals. Sex was used as a stratum in the randomization of the participants and thus included in the model to give a valid inference [34]. In addition to “sex,” another stratum used in the randomization process and included in the model was “rac,” a binary variable indicating whether a participant was randomly assigned as a couple or not. In case of heterogeneity, the model also included a variance function structure allowing for different variances per stratum of “group” and/or “time.” The A-HCLF group was predetermined as the comparator arm. Further details about the use of cLMMs have been previously described [35,36].

Values were transformed by the natural logarithm (ln) before analyses of responses in relative terms [35]. Relative within-group changes from baseline to follow-up and between-group differences are reported in the main text and tables as percentages calculated from the regression coefficients (i.e., the mean of log-ratios) by the formula 100 × (exp^estimate^ − 1). In the figures, however, we show results in relative terms as sympercents (s%), which are additive and symmetric percentage differences on the 100 log_e_ scale [37]. This relative measure is calculated as the difference between the natural logs of 2 numbers multiplied by 100, i.e.,100 × ln(a) – 100 × ln(b) = 100 × ln(a/b), making it straightforward to present and interpret without back transformation.

In a separate model (data not shown), we additionally adjusted for total energy, PUFA, and alcohol intake, as well as C-reactive protein and cotinine, which were factors we aimed to control for in the study design and were not expected to differ between groups at baseline or during the intervention. The results were largely unaffected by this additional adjustment (data not shown).

Linear mixed-effects models are regarded as an optimal approach in trials with repeated measurements where considerable missing data occur [[38], [39], [40]]. Therefore, we did not predefine another strategy to manage potential intermittent missing outcomes or data loss due to participant dropouts.

Data are presented as arithmetic means ± SD, unadjusted geometric means ± 1 SD ranges, or mean score differences {±relative effect estimates [95% confidence interval (CI)]} if not otherwise stated. The geometric SD ranges used in descriptive statistics were calculated by dividing and multiplying the geometric means by the geometric SD factors to obtain the lower and upper limits, respectively [35]. All inferential tests were 2-tailed with a nominal *α* level of 0.05. Because analyses of multiple metabolites and contrasts were exploratory, nominal *P* values are presented as the primary inferential metric [41], and statistical significance should therefore be interpreted cautiously. However, to account for testing across multiple metabolites, we additionally controlled the false discovery rate (FDR) using the Benjamini–Hochberg procedure for each prespecified between-group and within-group contrast at each follow-up. Corresponding FDR-adjusted *P* values (*q* values) are reported in the results text when nominal significance either remained or shifted after FDR correction, with further details provided in Supplementary Tables 1-4.

To assess the robustness of the primary ITT findings and address potential bias due to drop out, we performed sensitivity analyses adjusting for diet adherence and performed inverse probability weighting (IPW) analyses. The models adjusted for diet adherence repeated the primary cLMMs but included adjustment for self-reported adherence derived from the previously described self-reported adherence questionnaire, as well as deviations between planned and reported intake derived from repeated dietary records. Dietary deviations were calculated as the difference between planned and reported intake for total energy (kcal) and macronutrients (expressed as percentage of total energy intake), with baseline dietary deviations set to zero. In the IPW analysis, observations with available follow-up outcome data were weighted by the inverse of the estimated probability of having observed follow-up data. Stabilized weights were estimated using logistic regression models, with baseline covariates (group, sex, rac, and baseline outcome value) included in the numerator and both baseline covariates and time (visit) included in the denominator. The stabilized weights were incorporated through the weights argument in the lme function using a fixed variance structure [varFixed(∼sw)], thus including them in the estimation. The sensitivity analyses were compared with the primary ITT analyses to assess whether estimated between-group differences and nominal significance status were substantially altered.

To explore whether absolute changes in plasma B-vitamers correlated with absolute changes in estimated dietary B-vitamin intake, Spearman correlation analyses were performed using the package *psyc v.2.4.5.26.*

## Results

### Participant characteristics

After screening, a total of 193 participants were randomly assigned to 1 of the 3 study diets. After randomization, 1 participant withdrew consent, leaving available baseline data for 192 participants. A flow diagram of the study can be found in Supplementary Figure 1. Attrition increased over time, from 38% at 3 mo to 70% at 12 mo. Participants in the study had a mean age of 41.6 ± 8.8 y, and close to half of the participants were males. At baseline, for males and females, respectively, mean BMI was 36.5 ± 4.9 and 36.8 ± 4.7. Thirty participants (10 females) were categorized as smokers/snus users based on plasma cotinine values >85 μmol/L. Table 1 shows baseline plasma metabolite values, total dietary energy-, macronutrient-, and B-vitamin intakes for the whole study population and for males and females separately. Recorded energy intake at baseline was 2541 ± 567 kcal in males and 2111 ± 515 kcal in females (Table 1). By 12 mo, participants had lost between 5% and 7% body weight with no significant group differences, but after 6 mo, participants in the LCHF group had a greater reduction in body weight, waist-to-height ratio, and body fat mass, as reported previously [29].

**TABLE 1:** Baseline plasma metabolite concentrations and dietary energy, macronutrient, and B-vitamin intakes

Variable	All	Female	Male
Plasma metabolite^1^			
Methionine (μmol/L)	25.4 (22.7, 28.4)	25.4 (22.7, 28.4)	29.5 (25.8, 33.8)
tHcy (μmol/L)	8.92 (6.90, 11.5)	8.15 (6.23, 10.7)	9.87 (8.07, 12.1)
Cystathionine (μmol/L)	0.20 (0.13, 0.32)	0.18 (0.12, 0.27)	0.23 (0.15, 0.36)
Cysteine (μmol/L)	291 (261, 325)	278 (250, 309)	306 (280, 334)
α-hydroxybutyrate	50.1 (36.2, 69.3)	44.9 (33.5, 60.2)	56.6 (41.2, 77.7)
Choline (μmol/L)	9.15 (7.74, 10.8)	8.96 (7.51, 10.7)	9.38 (8.02, 11.0)
Betaine (μmol/L)	28.9 (22.4, 37.3)	27.4 (21.1, 35.5)	30.8 (24.4, 38.8)
DMG (μmol/L)	3.68 (2.75, 4.96)	3.45 (2.59, 4.60)	3.96 (2.98, 5.26)
Sarcosine (μmol/L)	1.13 (0.83, 1.54)	1.04 (0.76, 1.43)	1.24 (0.95, 1.63)
Glycine (μmol/L)	238 (193, 293)	246 (193, 315)	229 (197, 266)
Serine (μmol/L)	128 (111, 148)	130 (111, 151)	127 (112, 144)
Riboflavin (nmol/L)	15.4 (9.01, 26.5)	15.4 (8.89, 26.5)	15.5 (9.11, 26.5)
FMN (nmol/L)	7.32 (4.58, 11.7)	7.17 (4.65, 11.1)	7.48 (4.51, 12.4)
NAM (nmol/L)	218 (141, 337)	211 (141, 315)	226 (141, 362)
mNAM (nmol/L)	149 (98.4, 225)	148 (96.7, 226)	150 (100, 224)
PL (nmol/L)	13.5 (8.64, 21.3)	13.2 (7.95, 21.8)	14.0 (9.45, 20.6)
PLP (nmol/L)	32.8 (19.3, 55.7)	29.9 (17.1, 52.3)	36.3 (22.5, 58.7)
PA (nmol/L)	16.9 (11.0, 26.1)	16.5 (9.99, 27.1)	17.5 (12.5, 24.5)
Folate (nmol/L)	12.3 (7.50, 20.0)	12.2 (7.03, 21.2)	12.3 (8.11, 18.6)
Cobalamin (pmol/L)	262 (173, 398)	256 (156, 419)	270 (197, 369)
MMA (μmol/L)	0.13 (0.10, 0.17)	0.13 (0.10, 0.16)	0.14 (0.11, 0.18)
Dietary intake^2^			
Energy (kcal)	2315 (580)	2111 (515)	2541 (567)
Fat (E%)	39.3 (6.76)	38.6 (7.00)	40.1 (6.43)
Carbohydrates (E%)	39.6 (7.71)	40.6 (8.15)	38.5 (7.05)
Protein (E%)	17.8 (3.56)	17.5 (4.01)	18.1 (2.96)
Vitamin B-2 (mg)	1.36 (1.60)	1.26 (1.67)	1.50 (1.48)
Vitamin B-3 (mg)	13.8 (1.53)	12.4 (1.57)	15.7 (1.43)
Niacin equivalents (mg)	28.3 (1.43)	24.8 (1.42)	32.8 (1.35)
Vitamin B6 (mg)	1.23 (1.60)	1.17 (1.71)	1.31 (1.46)
Folate (μg)	201 (1.53)	192 (1.62)	211 (1.41)
Vitamin B-12 (μg)	5.09 (1.77)	4.43 (1.77)	5.93 (1.70)

Abbreviations: DMG, dimethylglycine; E%, energy percent; MMA, methylmalonic acid; mNAM, 1-methylnicotinamide; NAM, nicotinamide; PA, 4-pyridoxic acid; PL, pyridoxal; PLP, pyridoxal 5′-phosphate; tHcy, total homocysteine.

1 Values are geometric means (1-SD ranges). Baseline characteristics for all randomized participants with available metabolite data (*n* = 184, males: *n* = 87, females: *n* = 97) at baseline.

2 Values are arithmetic means (SDs). Baseline characteristics for all randomized participants, excluding 1 participant who withdrew consent (*n* = 192, males: *n* = 91, females: *n* = 101).

### Dietary intake

Changes from baseline in dietary intakes have been reported previously [29]. Recorded mean daily energy intake did not change significantly from baseline in the LCHF or A-HCLF diets, but a significant reduction of ∼11% was observed for the C-HCLF diet after 6 and 12 mo. Total energy intake differed significantly between the HCLF diets after 12 mo when the C-HCLF group showed a slightly greater reduction than the A-HCLF group [between-group difference in change scores (95% CI): −290 kcal (−562, −18.8), *P* = 0.036]. For females in the A-HCLF, C-HCLF, and LCHF groups, mean daily energy intakes ranged from 1923 to 1981 kcal/d at 3 mo, and from 1820 to 2064 kcal/d at 12 mo. For males, mean daily energy intakes ranged from 2316 to 2585 kcal/d at 3 mo, and from 2568 to 2844 kcal/d at 12 mo.

Carbohydrate intake in the LCHF group was reduced from 39 E% to 11 E% after 3 mo and increased stepwise to 15 E% after 12 mo, whereas the proportion of fat increased from 40 E% to 70 E% after 3 mo and changed to 66% after 12 mo. For the A-HCLF and C-HCLF groups, E% from carbohydrates and fat throughout the intervention were 41 E% to 43 E% and 36 E% to 38 E%, respectively, with no significant changes from baseline. Protein accounted for ∼16 E% to 17 E% across all groups throughout the study. Sugar intake contributed <1 E% following the LCHF- and C-HCLF diets, and 1 E% to 2 E% on the A-HCLF diet. For fiber, intake remained relatively stable from baseline and throughout the intervention for the LCHF group, with a mean intake of 17 to 19 g/d, whereas an increase from 19 to 21 g/d at baseline to 32 E% to 33 g/d and 38 E% to 43 g/d was observed for the A-HCLF and C-HCLF groups, respectively.

At 3 mo, self-reported adherence was similar across groups and sexes of ∼67% to 80%. However, a significant difference in change scores between the LCHF and A-HCLF groups was observed after 3 mo, as adherence was reported to be higher in the LCHF group (LCHF/A-HCLF: 80%/71%). As reported previously [42], high long-term adherence to the LCHF diet was further evident from highly sensitive plasma ketone measurements (GC-MS/MS), where the LCHF but not the HCLF diets showed over a 100% increase in plasma ketone concentrations (*β*-hydroxybutyrate and acetoacetate) from baseline to 3 mo, and over 75% increase at 6 and 9 mo, albeit without significant group differences at 12 mo [42].

### Between-group differences in plasma one-carbon metabolites and B-vitamers

**FIGURE 2:**
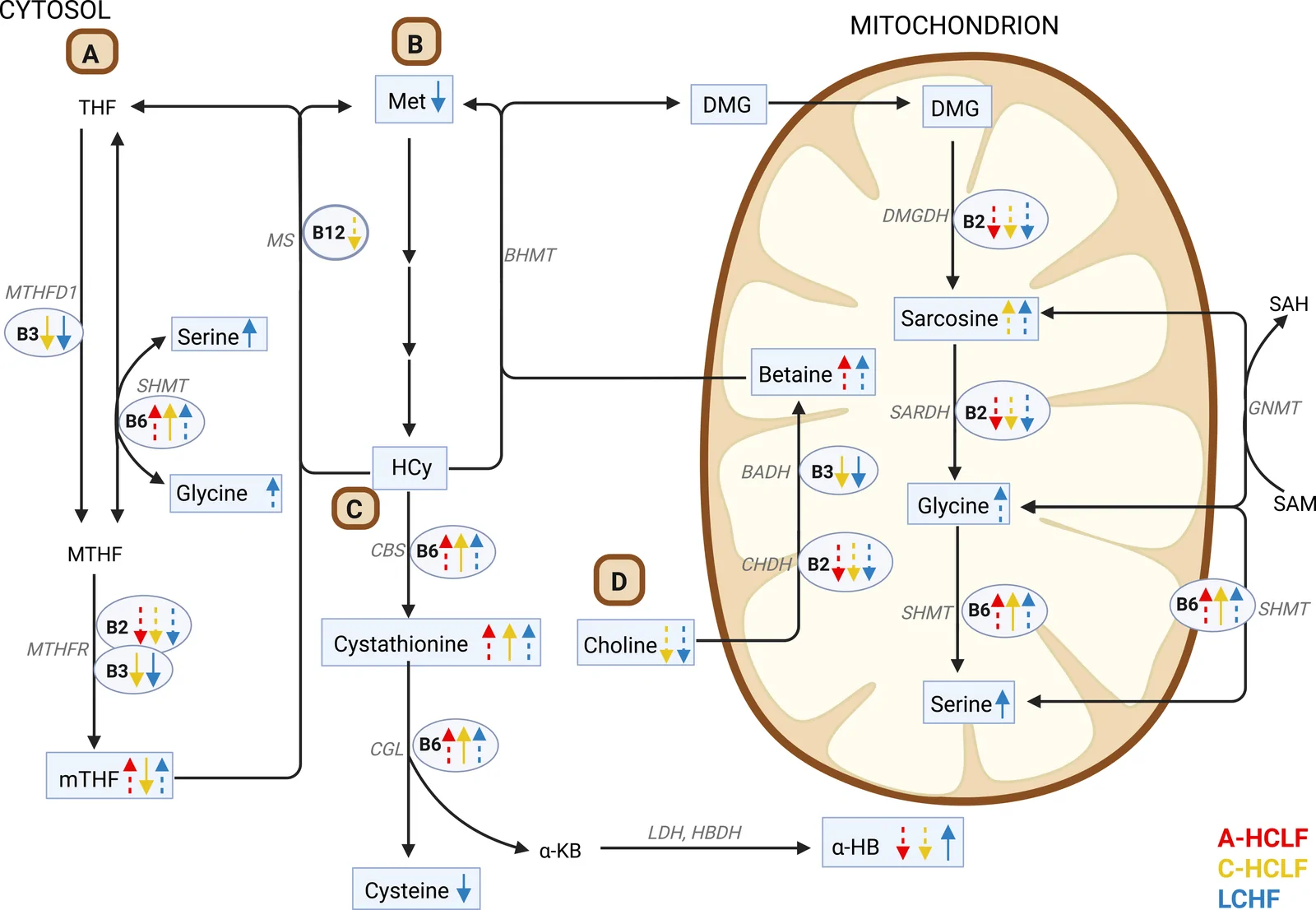
Pathway plot showing significant (*P* < 0.05) within-group changes and between-group differences in plasma one-carbon metabolites and B-vitamers throughout the intervention. (A) The folate cycle, (B) the methionine–homocysteine cycle, (C) the transsulfuration pathway, (D) the choline oxidation pathway. Relative model-adjusted within-group change scores from baseline to 3, 6, 9, and 12 mo, and the associated *P* values from the cLMMs laid the basis for the colored arrows indicating the direction of change (increase or decrease) within each group in the plot: A-HCLF (red), C-HCLF (yellow), LCHF (blue). The dashed arrows were superimposed on the plot when the observed within-group change from baseline was significant at least once throughout the intervention, whereas the solid arrows were superimposed on the plot when the observed between-group difference (C-HCLF or LCHF compared with A-HCLF) in change scores from baseline was significant at least once throughout the intervention. The direction of change for vitamin B-2 was based on the direction of change for plasma riboflavin, vitamin B-3 was based on plasma NAM and mNAM, and vitamin B-6 was based on the direction of change for plasma PLP. The squared boxes refer to plasma one-carbon metabolites measured in the current study, whereas circles indicate measured plasma B-vitamin cofactors. Text in italic refers to enzymes involved in the pathways. Created in BioRender. Bråtveit, M. (2026) https://BioRender.com/p32c4oj. α-HB, α-hydroxybutyrate; A-HCLF, acellular high-carbohydrate low-fat diet; α-KB, α-ketobutyrate; BADH, betaine aldehyde dehydrogenase; BHMT, betaine-homocysteine methyltransferase; CBS, cystathionine-β-synthase; CGL, cystathionine-γ-lyase; C-HCLF, cellular high-carbohydrate low-fat diet; CHDH, choline dehydrogenase; cLMM, constrained linear mixed-effects model; DMG, dimethylglycine; DMGDH, dimethylglycine dehydrogenase; GNMT, glycine-N-methyltransferase; HBDH, hydroxybutyrate dehydrogenase; Hcy, homocysteine; LDH, lactate dehydrogenase; LCHF, low-carbohydrate high-fat diet; Met, methionine; MS, methionine synthase; MTHF, 5,10-methylenetetrahydrofolate; MTHFD1, methylenetetrahydrofolate dehydrogenase complex 1; MTHFR, methylenetetrahydrofolate reductase; mTHF, 5-methyltetrahydrofolate; SAH, S-adenosylhomocysteine; SAM, S-adenosylmethionine; SARDH, sarcosine dehydrogenase; SHMT, serine hydroxymethyltransferase; THF, tetrahydrofolate.

**FIGURE 3:**
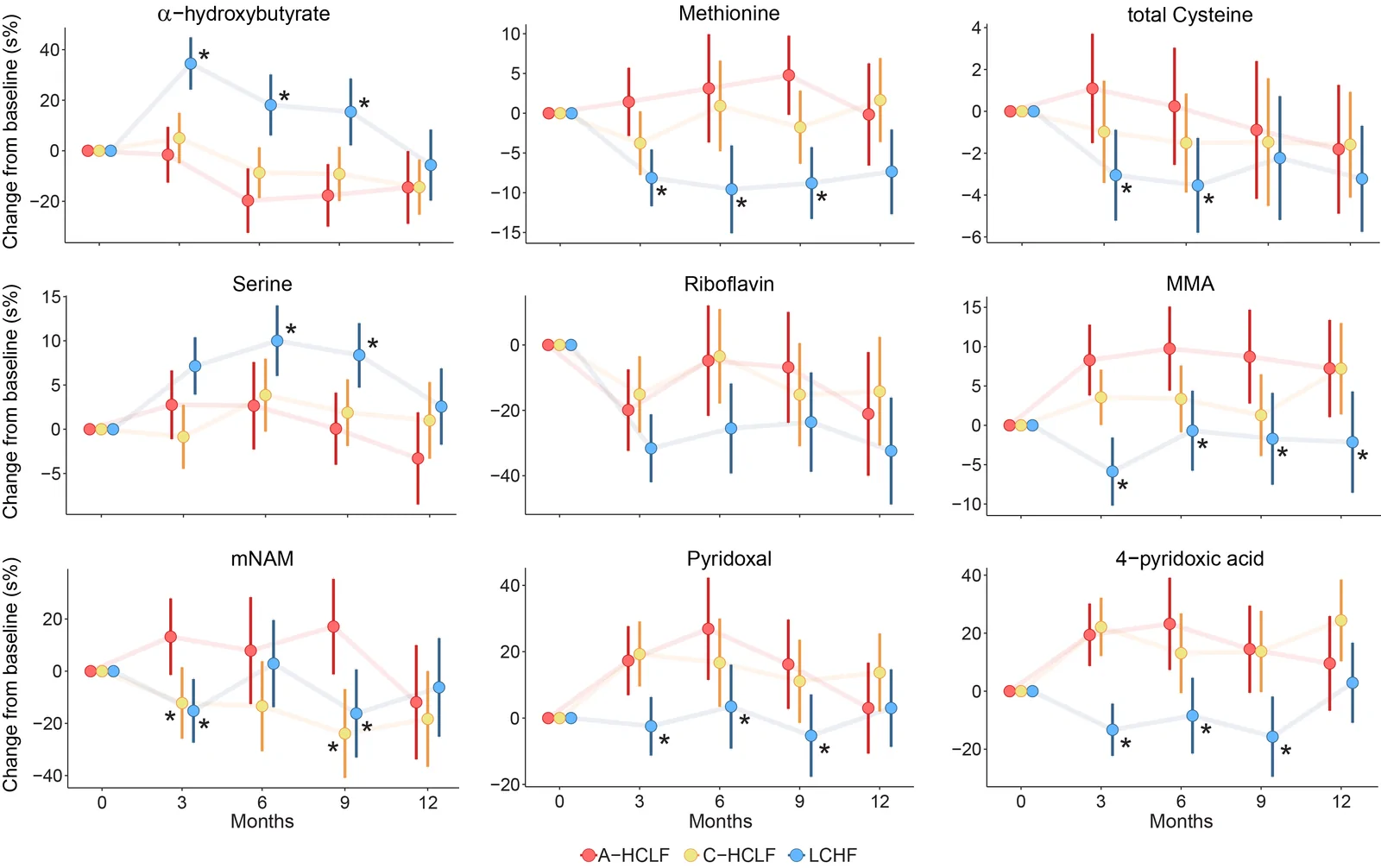
Relative changes from baseline of plasma metabolites and B-vitamers. Data were analyzed with constrained linear mixed-effects models (see main text). Before the analysis, values were transformed by the natural logarithm and multiplied by 100 to show relative changes as additive, symmetric percentages (sympercents; see main text). The asterisks superimposed on the plot indicate whether there was a significant between-group difference in either the LCHF or the C-HCLF group compared with the A-HCLF group after 3, 6, 9, and 12 mo of the intervention. These analyses were based on available metabolite data from 184 participants at baseline (A-HCLF, *n =* 65; C-HCLF, *n =* 59; LCHF, *n =* 60), 116 participants at 3 mo (A-HCLF, *n =* 32; C-HCLF, *n =* 37; LCHF, *n =* 47), 70 participants at 6 mo (A-HCLF, *n =* 17; C-HCLF, *n =* 26; LCHF, *n =* 27), 63 participants at 9 mo (A-HCLF, *n =* 19; C-HCLF, *n =* 22; LCHF, *n =* 22), and 57 participants at 12 mo (A-HCLF, *n =* 14; C-HCLF, *n =* 22; LCHF, *n =* 21). A-HCLF (red), acellular high-carbohydrate low-fat diet; C-HCLF (yellow), cellular high-carbohydrate low-fat diet; LCHF (blue), low-carbohydrate high-fat diet; MMA, methylmalonic acid; mNAM, 1-Methylnicotinamide; s%, sympercent.

Overall, we observed greater relative differences in plasma one-carbon metabolite and B-vitamer concentrations when comparing LCHF with the comparator A-HCLF diet than in the comparison of C-HCLF with A-HCLF (Figure 2). The methionine–homocysteine cycle, which governs methyl group transfer and remethylation, shifted at multiple nodes under the LCHF diet. For plasma methionine, we found a significant between-group difference in relative change scores after 3 mo (LCHF: −7.81%∗ compared with A-HCLF: +1.44%, *P* for between-group difference in relative change scores = 0.001; ∗, significant within-group change score), which persisted after 9 mo and after controlling for FDR (all *q* values < 0.05) as well as after adjustment for diet adherence, whereas no between-group differences were found for tHcy (Figure 3, Table 2, Supplementary Tables 1, 2 and 5). For folate and cobalamin, we found no significant between-group differences, although a significant between-group difference was found after 3 mo for the functional vitamin B-12 marker MMA (LCHF: −5.55%∗ compared with A-HCLF: +7.96%∗, *P* < 0.001), which persisted until 12 mo and after adjustment for diet adherence (Figure 3, Table 2, Supplementary Tables 2 and 3). After FDR adjustment, however, a shift in nominal significance was observed for MMA after 6 (*P =* 0.005, *q* = 0.067), 9 (*P =* 0.013, *q* = 0.062), and 12 (*P =* 0.037, *q* = 0.339) mo (Supplementary Table 1). The B3 vitamers, which reflect NAD/NADP-dependent redox metabolism, showed a significant between-group difference after 3 mo for mNAM (LCHF: −14.1%∗ compared with A-HCLF: +14.1%, *P* = 0.003, *q* = 0.018), which persisted through 9 mo (*P =* 0.007, *q* = 0.042), but not after adjusting for diet adherence (Supplementary Tables 1, 2 and 5). NAM showed a similar pattern although nominal significance at 9 mo shifted both after controlling for FDR (*q* = 0.145) and after adjustment for diet adherence (Figure 3, Table 2, Supplementary Tables 1, 2 and 5).

**TABLE 2:** Between-group differences in relative change scores from baseline for plasma one-carbon metabolites and B-vitamers after 3, 6, 9, and 12 mo^1^

	% difference after 3 mo vs. A-HCLF^2^	*P* value^2^	% difference after 6 mo vs. A-HCLF^3^	*P* value^3^	% difference after 9 mo vs. A-HCLF^4^	*P* value^4^	% difference after 12 mo vs. A-HCLF^5^	*P* value^5^
Methionine–homocysteine cycle metabolites (μmol/L)								
Methionine								
LCHF	–9.12 (–13.9, –4.06)	0.001	–11.9 (–19.2, –3.99)	0.004	–12.7 (–18.3, –6.70)	<0.001	–6.94 (–14.3, 1.07)	0.088
C-HCLF	–5.06 (–10.3, 0.52)	0.074	–2.19 (–10.4, 6.75)	0.618	–6.33 (–12.4, 0.15)	0.055	1.83 (–6.22, 10.6)	0.666
tHcy								
LCHF	–1.17 (–7.16, 5.21)	0.711	2.52 (–5.43, 11.1)	0.545	–3.86 (–12.8, 6.00)	0.428	1.15 (–7.70, 10.8)	0.806
C-HCLF	–0.059 (–6.21, 6.49)	0.985	0.12 (–7.41, 8.27)	0.976	–2.74 (–11.4, 6.78)	0.559	2.65 (–5.66, 11.7)	0.542
Transsulfuration pathway−related metabolites (μmol/L)								
Cystathionine								
LCHF	30.7 (–2.49, 75.3)	0.073	17.9 (–8.30, 51.7)	0.198	–7.42 (–34.2, 30.2)	0.657	1.83 (–26.6, 41.2)	0.913
C-HCLF	10.1 (–18.1, 48.0)	0.522	33.8 (7.10, 67.1)	0.011	1.38 (–26.2, 39.2)	0.932	4.52 (–22.4, 40.7)	0.770
Cysteine								
LCHF	–4.06 (–7.21, –0.80)	0.015	–3.71 (–7.08, –0.22)	0.038	–1.33 (–5.55, 3.08)	0.548	–1.40 (–5.20, 2.56)	0.482
C-HCLF	–2.05 (–5.44, 1.46)	0.247	–1.74 (–5.24, 1.89)	0.341	–0.57 (–4.88, 3.94)	0.799	0.22 (–3.63, 4.23)	0.911
α-hydroxybutyrate								
LCHF	43.5 (24.6, 65.3)	<0.001	46.0 (23.2, 73.1)	<0.001	39.2 (16.7, 66.0)	<0.001	9.29 (–10.4, 33.3)	0.379
C-HCLF	6.80 (–7.07, 22.8)	0.353	11.7 (–4.55, 30.7)	0.167	8.86 (–7.12, 27.6)	0.294	0.10 (–16.3, 19.7)	0.991
Choline oxidation pathway metabolites (μmol/L)								
Choline								
LCHF	–5.25 (–12.0, 1.97)	0.149	–4.10 (–12.5, 5.16)	0.373	–6.19 (–13.7, 2.01)	0.134	–6.31 (–13.9, 1.95)	0.130
C-HCLF	–3.85 (–11.0, 3.89)	0.319	–7.57 (–15.8, 1.51)	0.099	0.44 (–7.65, 9.25)	0.918	–6.98 (–14.5, 1.26)	0.095
Betaine								
LCHF	–1.24 (–8.98, 7.16)	0.763	–5.41 (–14.6, 4.78)	0.286	7.37 (–3.77, 19.8)	0.203	–8.91 (–19.4, 2.90)	0.133
C-HCLF	–5.76 (–13.5, 2.71)	0.176	–8.26 (–17.3, 1.83)	0.105	–0.69 (–11.0, 10.9)	0.902	–9.75 (–20.0, 1.85)	0.096
DMG								
LCHF	–2.96 (–12.2, 7.22)	0.554	–4.31 (–16.5, 9.68)	0.526	3.80 (–8.57, 17.8)	0.564	–6.96 (–19.2, 7.09)	0.313
C-HCLF	–9.41 (–18.5, 0.64)	0.065	–9.08 (–20.9, 4.50)	0.179	–2.64 (–14.3, 10.7)	0.681	–8.00 (–20.1, 5.87)	0.243
Glycine								
LCHF	1.38 (–3.81, 6.84)	0.608	2.60 (–6.22, 12.3)	0.575	2.46 (–4.61, 10.0)	0.504	2.28 (–3.61, 8.54)	0.455
C-HCLF	–3.42 (–8.61, 2.08)	0.218	–3.20 (–11.7, 6.08)	0.485	–0.62 (–7.58, 6.85)	0.865	1.06 (–4.84, 7.33)	0.729
Serine								
LCHF	4.48 (–0.59, 9.80)	0.084	7.64 (1.11, 14.6)	0.021	8.67 (2.94, 14.7)	0.003	6.04 (–0.80, 13.4)	0.085
C-HCLF	–3.55 (–8.48, 1.64)	0.175	1.21 (–5.02, 7.84)	0.710	1.83 (–3.61, 7.58)	0.516	4.40 (–2.35, 11.6)	0.206
Sarcosine								
LCHF	1.39 (–8.44, 12.3)	0.791	3.64 (–8.69, 17.6)	0.579	5.88 (–3.64, 16.3)	0.234	–1.98 (–13.7, 11.3)	0.757
C-HCLF	–4.32 (–14.1, 6.54)	0.420	–2.03 (–13.9, 11.4)	0.754	0.56 (–8.50, 10.5)	0.907	–6.02 (–17.2, 6.64)	0.335
B-vitamers								
B2 vitamers (nmol/L)								
Riboflavin								
LCHF	–11.0 (–24.1, 4.32)	0.150	–18.7 (–34.6, 0.91)	0.060	–15.4 (–32.6, 6.13)	0.148	–10.8 (–30.4, 14.4)	0.368
C-HCLF	4.91 (–11.3, 24.1)	0.575	1.33 (–18.8, 26.4)	0.907	–8.06 (–27.0, 15.8)	0.475	7.12 (–16.6, 37.6)	0.589
FMN								
LCHF	1.31 (–10.3, 14.4)	0.833	–4.65 (–17.3, 9.89)	0.510	9.81 (–4.48, 26.2)	0.187	0.81 (–18.6, 24.9)	0.941
C-HCLF	–4.17 (–15.4, 8.59)	0.503	2.45 (–9.76, 16.3)	0.708	3.19 (–8.84, 16.8)	0.618	4.96 (–15.5, 30.4)	0.661
B3 vitamers (nmol/L)								
NAM								
LCHF	–29.6 (–41.9, –14.6)	<0.001	–4.63 (–30.0, 29.9)	0.763	–23.3 (–40.6, –0.87)	0.043	5.86 (–20.9, 41.6)	0.700
C-HCLF	–22.4 (–35.4, –6.70)	0.007	–18.3 (–39.3, 9.95)	0.181	–14.8 (–32.5, 7.56)	0.177	–1.81 (–24.6, 27.8)	0.892
mNAM								
LCHF	–24.7 (–37.6, –9.28)	0.003	–4.86 (–26.6, 23.4)	0.706	–28.3 (–43.6, –8.78)	0.007	5.86 (–20.5, 41.0)	0.696
C-HCLF	–22.4 (–36.3, –5.54)	0.012	–19.2 (–37.9, 5.21)	0.113	–33.6 (–47.8, –15.5)	0.001	–6.24 (–29.3, 24.4)	0.655
B6 vitamers (nmol/L)								
PLP								
LCHF	–9.69 (–21.1, 3.35)	0.138	–16.1 (–32.6, 4.45)	0.116	–14.7 (–31.8, 6.59)	0.161	–0.15 (–15.7, 18.3)	0.986
C-HCLF	4.47 (–9.37, 20.4)	0.545	–6.76 (–25.5, 16.6)	0.539	–6.94 (–25.7, 16.6)	0.530	20.2 (1.59, 42.3)	0.032
PL								
LCHF	–17.9 (–27.9, –6.65)	0.003	–20.9 (–35.1, –3.63)	0.020	–19.4 (–32.3, –3.99)	0.016	0.013 (–16.1, 19.2)	0.999
C-HCLF	2.05 (–10.9, 16.9)	0.768	–9.71 (–26.2, 10.5)	0.321	–5.03 (–20.3, 13.2)	0.563	11.3 (–6.66, 32.8)	0.232
PA								
LCHF	–27.9 (–37.0, –17.4)	<0.001	–27.1 (–40.6, –10.6)	0.002	–26.0 (–39.4, –9.77)	0.003	–6.45 (–24.3, 15.5)	0.535
C-HCLF	2.74 (–10.9, 18.5)	0.709	–9.60 (–26.6, 11.3)	0.341	–0.79 (–18.8, 21.2)	0.938	16.0 (–6.22, 43.5)	0.171
Folate (nmol/L)								
LCHF	5.58 (–9.23, 22.8)	0.480	–15.0 (–31.7, 5.82)	0.145	16.0 (–9.89, 49.2)	0.249	–9.57 (–28.4, 14.1)	0.396
C-HCLF	–10.2 (–24.7, 7.00)	0.228	–22.1 (–39.0, –0.51)	0.045	–12.9 (–33.8, 14.6)	0.323	–18.6 (−37.0, 5.22)	0.116
Cobalamin (pmol/L)								
LCHF	3.56 (–5.39, 13.3)	0.447	4.61 (–6.48, 17.0)	0.429	10.2 (–1.75, 23.6)	0.097	–1.02 (–13.5, 13.2)	0.880
C-HCLF	–4.63 (–13.4, 5.04)	0.335	–6.59 (–16.9, 4.97)	0.251	0.78 (–10.5, 13.5)	0.898	2.29 (–10.9, 17.4)	0.748
MMA (μmol/L)								
LCHF	–13.2 (–18.4, –7.68)	<0.001	–9.91 (–16.2, –3.14)	0.005	–9.90 (–17.0, –2.22)	0.013	–8.95 (–16.6, –0.57)	0.037
C-HCLF	–4.62 (–9.90, 0.96)	0.103	–6.19 (–12.3, 0.37)	0.064	–7.16 (–14.1, 0.37)	0.062	–0.031 (–8.11, 8.76)	0.994

Abbreviations: A-HCLF, acellular high-carbohydrate low-fat; C-HCLF, cellular high-carbohydrate low-fat; CI, confidence interval; cLMM, constrained linear mixed-effects model; DMG, dimethylglycine; LCHF, low-carbohydrate high-fat; MMA, methylmalonic acid; mNAM, 1-Methylnicotinamide; NAM, nicotinamide; PA, 4-pyridoxic acid; PL, pyridoxal; PLP, pyridoxal 5′-phosphate; tHcy, total homocysteine.

1 Fasting plasma levels of one-carbon metabolites and B-vitamers were analyzed with cLMMs. Values were transformed by the natural logarithm before the analyses. The A-HCLF diet group was defined as the comparator in the between-group comparisons. The between-group differences in relative change scores from baseline to follow-up are reported as percentages (95% CIs) calculated from cLMM estimates: % = (exp^estimate^ − 1) × 100.

2 Relative model-adjusted between-group differences after 3 mo and *P* values from the cLMM. At 3 mo, data from a total of 116 participants were available (A-HCLF, *n =* 32; C-HCLF, *n =* 37; LCHF, *n =* 47).

3 Relative model-adjusted between-group differences after 6 mo and *P* values from the cLMMs. At 6 mo, data from a total of 70 participants were available (A-HCLF, *n =* 17; C-HCLF, *n =* 26; LCHF, *n =* 27).

4 Relative model-adjusted between-group differences after 9 mo and *P* values from the cLMMs. At 9 mo, data from a total of 63 participants were available (A-HCLF, *n =* 19; C-HCLF, *n =* 22; LCHF, *n =* 22).

5 Relative model-adjusted between-group differences after 12 mo and *P* values from the cLMMs. At 12 mo, data from a total of 57 participants were available (A-HCLF, *n =* 14; C-HCLF, *n =* 22; LCHF, *n =* 21).

Downstream, the transsulfuration pathway, which converts homocysteine to cysteine and depends on PLP (B6) for cystathionine-β-synthase (CBS)/cystathionine-*γ*-lyase activity, also appeared to be affected by the LCHF diet. α-HB showed a significant between-group difference after 3 mo (LCHF: +41.3%∗ compared with A-HCLF: −1.56%, *P* < 0.001), with differences sustained through 9 mo, and also after FDR correction and adjustment for diet adherence (Figure 3, Table 2, Supplementary Tables 1, 2 and 5). Cysteine showed a small but significant between-group difference after 3 mo (LCHF: −3.00%∗ compared with A-HCLF: +1.10%, *P* = 0.015), which remained significant after 6 mo, but not after FDR correction (*P =* 0.038, *q* = 0.275) or adjustment for diet adherence (Figure 3, Table 2, Supplementary Tables 1, 2 and 5). Concordantly, plasma B6 vitamer concentrations shifted, resulting in significant between-group differences for PL after 3 mo (LCHF: −2.42% compared with A-HCLF: +18.9%∗, *P* = 0.003) with a similar pattern observed for PA (LCHF: −12.4%∗ compared with A-HCLF: +21.4%∗, *P* < 0.001), which persisted after 9 mo (Figure 3, Table 2, Supplementary Table 2). However, after controlling for FDR, the differences for PL at 6 mo (*P =* 0.020, *q* = 0.176) and 9 mo (*P =* 0.016, *q* = 0.075), as well as for PA at 6 mo (*P =* 0.002, *q* = 0.054), shifted in nominal significance (Supplementary Table 1). Adjustment for diet adherence also changed the nominal significance results. For PL, the observed differences were no longer significant at any time point, whereas for PA the difference remained significant after 3 mo but not after 6 or 9 mo (Supplementary Table 5). Along the choline oxidation pathway, we found significant between-group differences for serine after 6 (LCHF: +10.5%∗ compared with A-HCLF: +2.69%, *P* = 0.021) and 9 mo (LCHF: +8.74%∗ compared with A-HCLF: +0.062%, *P* = 0.003), although the difference at 6 mo shifted in nominal significance after FDR correction (*q* = 0.176) (Figure 3, Table 2, Supplementary Table 1). When adjusting for dietary adherence, however, significance remained after 6 mo, but not after 9 mo (Supplementary Table 5).

By contrast, among participants on the C-HCLF diet, methionine and tHcy did not change or differ from the A-HCLF diet, and choline pathway metabolites were similarly unchanged (Figure 3, Table 2, Supplementary Table 2). Along the transsulfuration pathway, cystathionine—the CBS-dependent intermediate upstream of cysteine—showed a significant between-group difference in relative change scores after 6 mo (C-HCLF: +37.1%∗ compared with A-HCLF: +2.51%, *P* for between-group difference in relative change scores = 0.011; ∗, significant within-group change score), without downstream propagation (Figure 3, Table 2, Supplementary Table 2). However, this difference was not significant after FDR correction (*q* = 0.463) or adjustment for diet adherence (Supplementary Tables 1 and 5). For cofactors, significant between-group differences were found for PLP (active vitamin B-6) after 12 mo (C-HCLF: +38.3%∗ compared with A-HCLF: +15.0%, *P* = 0.032), and folate after 6 mo (C-HCLF: −1.80% compared with A-HCLF: +26.0%, *P* = 0.045), both remaining significant after adjustment for diet adherence but not after FDR correction (Supplementary Tables 1, 2 and 5). No between-group differences were found for cobalamin or MMA (Figure 3 and Table 2). Thus, when compared with A-HCLF, the C-HCLF diet appeared to primarily reduce folate, increase PLP, and transiently increase cystathionine, whereas LCHF induced broader and more sustained alterations across interconnected nodes of one-carbon metabolism and its vitamin cofactor milieu.

### Associations between dietary intake of B-vitamins and plasma B-vitamers

**FIGURE 4:**
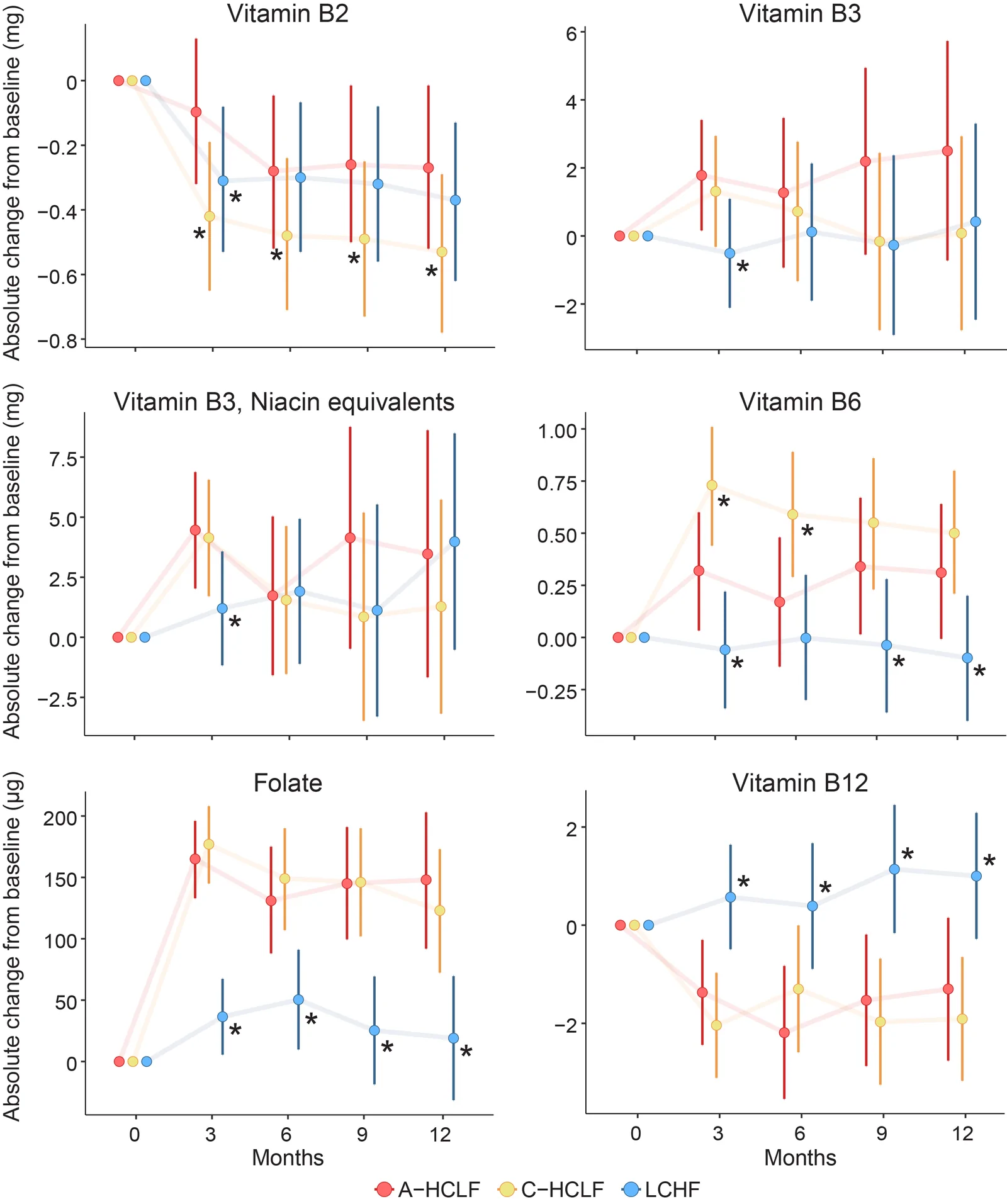
Absolute changes from baseline in dietary B-vitamin intake during the intervention. The red color indicates the A-HCLF group, the yellow color indicates the C-HCLF group, and the blue color indicates the LCHF group. The asterisks superimposed on the plot indicate whether there was a significant between-group difference from baseline in either the LCHF or the C-HCLF group compared with the A-HCLF group after 3, 6, 9, and 12 mo of the intervention. The analyses of vitamin intake were based on available vitamin intake data from 163 participants at 3 mo (A-HCLF, *n =* 53; C-HCLF, *n =* 53; LCHF, *n =* 57), 82 participants at 6 mo (A-HCLF, *n =* 23; C-HCLF, *n =* 29; LCHF, *n =* 30), 63 participants at 9 mo (A-HCLF, *n =* 19; C-HCLF, *n =* 23; LCHF, *n =* 21), and 52 participants at 12 mo (A-HCLF, *n =* 12; C-HCLF, *n =* 21; LCHF, *n =* 19). A-HCLF (red), acellular high-carbohydrate low-fat diet; C-HCLF (yellow), cellular high-carbohydrate low-fat diet; LCHF (blue), low-carbohydrate high-fat diet.

We next assessed whether the intake of B-vitamins throughout the intervention contributed to the group-specific changes in plasma marker levels. Overall, dietary intake of B-vitamins differed significantly between groups throughout the intervention, with the LCHF group showing lower intakes of vitamins B-2, B-3, B-6, and folate, compared with the A-HCLF group, but higher intake of vitamin B-12, whereas the C-HCLF group consistently had the highest intake of vitamin B-6 (Figure 4).

Intake of vitamin B-2 was reduced from baseline in all groups, with the largest reduction in the C-HCLF group, which was significantly different from the A-HCLF comparator group throughout the whole intervention, although shifting in nominal significance at 9 (*P =* 0.012, *q* value = 0.103) and 12 (*P =* 0.005, *q* = 0.076) mo when controlling for FDR (Figure 4, Supplementary Tables 3 and 4). Between-group differences were also observed after 3 mo when comparing LCHF with A-HCLF (LCHF: −0.31 mg∗ compared with A-HCLF: −A-10 mg, *P* for between-group difference < 0.001; ∗, significant within-group change score), but this difference attenuated throughout the intervention because the intake of vitamin B-2 was similarly reduced in the A-HCLF group by 6 mo and onward (Figure 4, Supplementary Tables 3 and 4). For niacin, a modest decrease in LCHF and an increase in A-HCLF contributed to a significant group difference at 3 mo (LCHF: −0.51 mg compared with A-HCLF: +1.78 mg∗, *P =* 0.005), whereas intake of niacin equivalents was significantly different because of a greater increase in the A-HCLF by 3 mo (LCHF: +1.20 mg compared with A-HCLF: +4.46 mg∗, *P* = 0.018), both remaining significant also after FDR correction (Figure 4, Supplementary Tables 3 and 4). Vitamin B6 intake increased from baseline in both HCLF groups and remained increased compared with baseline throughout the intervention, but with a larger increase for C-HCLF at 3 (C-HCLF: +0.73 mg∗ compared with A-HCLF: +0.32 mg∗, *P <* 0.001) and 6 mo (C-HCLF: +0.59 mg∗ compared with A-HCLF: +0.17 mg, *P* = 0.002), both of which remained significant also after FDR correction (Figure 4, Supplementary Tables 3 and 4). Vitamin B-6 intake in the LCHF group was slightly reduced, with significant between-group differences both after 3 (LCHF: −0.06 mg compared with A-HCLF: +0.32∗ mg, *P <* 0.001), 9 (LCHF: −0.4 mg compared with A-HCLF: +0.34 mg∗, *P* = 0.020), and 12 (LCHF: −0.10 mg compared with A-HCLF: +0.31 mg, *P* = 0.007) mo, which remained significant after controlling for FDR (Figure 4, Supplementary Tables 3 and 4). The intake of folate increased similarly in both HCLF groups, but less in the LCHF group, resulting in between-group differences after 3 mo (LCHF: +36.5 μg∗ compared with A-HCLF: +165 μg∗, *P <* 0.001), which remained significant throughout the intervention and after FDR correction (Figure 4, Supplementary Tables 3 and 4). On the other hand, the intake of vitamin B-12 increased from baseline in the LCHF group while decreasing in both HCLF groups, resulting in significant between-group differences after 3 mo (LCHF: +0.57 μg compared with A-HCLF: −A-H7 μg∗, *P <* 0.001), which remained throughout the intervention and after controlling for FDR (Figure 4, Supplementary Tables 3 and 4).

Despite significant changes in both dietary B-vitamin intakes and plasma B-vitamer concentrations, there were no modest or strong correlations between these measures in any of the groups (Supplementary Figures 2–4). In the LCHF group, the strongest correlations were observed for changes in estimated dietary folate intake and changes in plasma folate (Spearman’s rho, *r*_s_ = 0.29, *P =* 0.051) and for changes in estimated dietary vitamin B-6 intake and changes in plasma PLP (*r*_s_ = 0.29, *P =* 0.049). For A-HCLF, the strongest correlation was found between changes in dietary vitamin B-12 intake and changes in plasma cobalamin (*r*_s_ = 0.30, *P* = 0.090), whereas for C-HCLF only negligible correlations were observed.

### Sensitivity analyses

In general, adjustment for diet adherence did not substantially alter the interpretation of the primary findings, although some changes in nominal significance were observed in the comparison of LCHF with A-HCLF, such as for cysteine, the B3-vitamers (NAM and mNAM), and B6-vitamers (PL and PA), as noted above (Supplementary Table 5). Adjustment for diet adherence also led to a change in nominal significance for riboflavin, which became significant in the LCHF compared with A-HCLF comparison at 3, 6, and 9 mo. In the IPW sensitivity analysis applied to assess robustness to potential dropout-related bias, we found only 1 change in nominal significance status for the plasma metabolites. This was for cystathionine in the comparison of C-HCLF compared with A-HCLF after 6 mo, changing from significant in the primary ITT analysis (*P =* 0.011) to nonsignificant in the IPW analysis (*P =* 0.053) (Supplementary Table 6). For dietary B-vitamin intakes, we observed no differences in nominal significance between the primary ITT and the IPW sensitivity analysis (Supplementary Table 7).

## Discussion

The present secondary exploratory analyses of the CARBFUNC randomized controlled trial contribute to a deeper understanding of diet-induced metabolic adaptations. We observed pronounced alterations in plasma one-carbon metabolites in the LCHF group, including higher α-HB and serine, and lower total cysteine, methionine, MMA, NAM, mNAM, PL, and PA compared with the A-HCLF comparator group. Notably, the greatest within-group changes from baseline and between-group differences were observed after 3 and 6 mo of follow-up, when participant retention and adherence overall remained relatively high, whereas the more substantial attrition by 9 and 12 mo warrants more cautious interpretation. Generally, differences in the estimated dietary intake of the B-vitamins could not explain the changes in their respective plasma markers. Thus, our findings suggest that macronutrient composition, independent of dietary B-vitamin intake, elicits strong metabolic changes in one-carbon metabolism, whereas carbohydrate quality—at least by carbohydrate “cellularity” as implemented in the present study, with minimal sugar intake—has less impact. This study provides novel insights into how macronutrient composition, particularly carbohydrate restriction, can elicit marked responses in one-carbon metabolism that likely reflect adaptive shifts to ketosis, and highlights the importance of considering dietary context when interpreting metabolite markers linked to metabolic health and disease risk.

Although we acknowledge that plasma metabolite concentrations—in the absence of tissue-specific or flux data—cannot identify which tissues underlie the changes we observed, the overall changes, particularly in the LCHF group, suggest altered one-carbon substrate flux and add to the growing body of knowledge on the metabolic effects of ketogenic diets. Methionine, the essential amino acid and precursor to the universal methyl donor SAM, was significantly reduced within the LCHF group, whereas tHcy levels remained stable. Because protein intake was similar across all groups, reduced dietary intake is unlikely to explain the decline in plasma methionine. Another more plausible explanation could be that the LCHF diet resulted in sufficient SAM and energy (ATP) levels, causing homocysteine to be diverted from remethylation to methionine toward the transsulfuration pathway (Figure 1). This pathway irreversibly removes homocysteine as a mechanism to prevent SAM accumulation and/or to provide cysteine, a precursor for GSH and other sulfur-containing compounds involved in antioxidant defense and detoxification (Figure 1). This interpretation is supported by the elevated plasma levels of the transsulfuration metabolites cystathionine and α-HB [derived from α-ketobutyrate (α-KB), a byproduct of cystathionine conversion to cysteine], alongside the reduced cysteine concentrations.

The physiological purpose of this shift could be to enhance antioxidant capacity or sulfur metabolism under ketogenic conditions, consistent with the findings from Mardinoglu et al. [24], who reported increased de novo GSH synthesis after a short-term carbohydrate-restricted diet. In contrast, the livers of C57BL/6J mice subjected to a fatty liver–promoting high-fat high-sugar “Western” diet showed reduced GSH levels and downregulated transsulfuration genes, alongside upregulation of remethylation genes, including those involved in choline oxidation [43]. Contrary to a high-fat ketogenic diet, which, from our data, appears to enhance transsulfuration and antioxidant defense, the Western-type diet imposes metabolic stress that increases methylation demand for hepatic phosphatidylcholine synthesis required for VLDL export [44]. This stress drives a compensatory response to prevent further hepatic lipid accumulation, favoring remethylation of homocysteine to conserve methionine at the expense of transsulfuration. As a result, synthesis of cysteine and GSH is limited, compromising antioxidant capacity and reflecting a trade-off that may indicate a less adaptive state in the context of fatty liver than on LCHF diets, which are well documented to prevent hepatic lipid accumulation [45,46].

The observed increase in plasma α-HB, not to be confused with the major circulating ketone body *β*-hydroxybutyrate that rises during ketosis, following the ketogenic LCHF diet may reflect increased conversion from its precursor α-KB, which is generated as a byproduct of glutathione anabolism (transsulfuration pathway) and amino acid catabolism (methionine and threonine). As shown in Figure 1, α-KB can also be diverted toward the tricarboxylic acid cycle via conversion to propionyl-CoA, then methylmalonyl-CoA, and ultimately succinyl-CoA. However, when α-KB synthesis exceeds its catabolic rate, or when the enzyme responsible for the conversion to propionyl-CoA is inhibited—for example, by a high NADH/NAD^+^ ratio—α-HB is found to accumulate [47,48]. Interestingly, elevated α-HB has been proposed as a biomarker of insulin resistance [49], where its increase is linked to higher glycolytic and lipid flux, oxidative stress, and upregulated glutathione synthesis [50,51]. However, evidence suggests that the α-HB increase during ketosis may reflect a distinct mechanism. In rats fed a ketogenic diet (75E% from fat) for 3 wk, mitochondrial GSH levels and GSH/glutathione disulfide activity increased compared with controls, accompanied by improved redox status, reduced hydrogen peroxide production, and less mitochondrial DNA damage [49]. These findings suggest that α-HB elevation in the ketogenic state may represent protective adaptations rather than harmful processes. Consistent with this, our observed increase in plasma α-HB aligns with findings from other fasting (36–58 h and 6 d) and very-low-calorie studies (8 wk) [[50], [51], [52], [53]]. Moreover, the combination of elevated α-HB with reduced methionine observed in our study was also seen in a 36-h fasting study in healthy individuals, where both metabolites were proposed as potential markers of fasting [52]. However, because we observed comparable changes in participants following an LCHF diet over a much longer period, these markers are unlikely to reflect fasting per se. Instead, their combined changes may indicate shared metabolic adaptations to both fasting and ketogenic diets, such as enhanced fatty acid oxidation and improved resistance to oxidative stress. Accordingly, our results indicate that α-HB may not be a relevant indicator of insulin resistance in ketogenic contexts.

The observed increase in plasma serine following the LCHF diet could reflect a metabolic feedback response to altered nutrient availability. Serine is a gluconeogenic amino acid essential for various cellular functions, including its role as a major one-carbon unit donor in the folate-dependent remethylation cycle [54]. Although serine can be obtained through dietary intake, primarily from protein sources [55], it is unlikely that dietary protein intake explains the elevated plasma serine concentrations associated with the LCHF diet because protein intake was similar to the A-HCLF comparator arm and did not change significantly from baseline. However, serine can be produced endogenously via interconversion with glycine or through de novo synthesis from 3-phosphoglycerate, which is derived directly from glucose or indirectly from gluconeogenic precursors such as pyruvate. Notably, ∼75% of circulating serine in humans is synthesized de novo and released from the kidney during fasting, with pyruvate serving as the primary carbon source [54,56]. Although we cannot exclude that interconversion with glycine contributed to the serine increase on the LCHF diet, a greater contribution may have come from increased serine synthesis from gluconeogenic substrates, because an LCHF diet mimics a fasting state by increasing processes such as fatty acid oxidation and gluconeogenesis. This aligns with findings from a mouse study, where a ketogenic diet, both with and without protein restriction, was found to upregulate serine synthesis genes in the liver and cerebral cortex [57]. Taken together, the rise in serine along with signs of enhanced transsulfuration and cysteine utilization in the LCHF group may reflect metabolic adaptation to the ketogenic LCHF diet. This is consistent with a study in cancer models demonstrating that deprivation of glucose-induced serine biosynthesis, a response mediated by c-Myc that supports GSH production, nucleotide synthesis, and cell cycle progression—all key adaptations for survival under glucose-deprived conditions [58].

The observed plasma reduction in the vitamin B-3–metabolite NAM and its downstream catabolite mNAM in both the LCHF and C-HCLF groups compared with the A-HCLF comparator arm may reflect distinct metabolic changes driven by changes in macronutrient availability and carbohydrate quality. NAM is a precursor for, and the main source of, NAD^+^ [59]. NAD^+^ serves as an essential cofactor for several enzymes in one-carbon metabolism and as an electron carrier in key metabolic reactions, including *β*-oxidation and the tricarboxylic acid cycle. Reactions that require NAD^+^ yield NAM, which can be recycled to NAD^+^ again through the salvage pathway or excreted through mNAM [59]. The reduction in NAM on the LCHF diet could be expected, as ketone-dependent metabolism involves lower NAD^+^ turnover than glucose-dependent metabolism [60]. Lower NAD^+^ turnover may have reduced the generation of NAM from NAD^+^ breakdown and elevated the NAD^+^/NADH ratio. The resulting increase in NAD^+^ availability due to reduced NAD^+^ turnover might, in turn, have diminished the need for NAD^+^ salvage from NAM and suppressed the methylation to mNAM, leading to lower circulating levels of both metabolites. In contrast, the mechanism behind the difference between the A-HCLF and C-HCLF diets in NAM and mNAM is more difficult to interpret, but it is interesting that aspects of carbohydrate quality may also influence NAD^+^ turnover, possibly involving more rapid recycling of NAM and reduced excretion via mNAM.

Also, for vitamin B-6, our results suggest that the observed differences between LCHF and A-HCLF were driven by distinct metabolic changes resulting from differences in macronutrient intake rather than in dietary vitamin B-6 intake, because correlations between plasma cofactors and dietary intake were weak. Vitamin B-6 circulates mainly as PLP (active coenzyme), PL (transport intermediate), and PA (catabolic product) [61,62]. On the LCHF diet, only PA decreased markedly, contrasting with increased PL and PA on the comparator A-HCLF diet. This pattern could suggest that LCHF promotes greater tissue utilization, recycling, and reduced catabolic loss of vitamin B-6, likely reflecting increased vitamin B-6–dependent amino acid catabolism through transamination and enhanced flux via the vitamin B-6–dependent transsulfuration pathway during ketoadaptation. These metabolic shifts further align with the observed decreases in methionine and MMA and the rise in α-HB. In contrast, higher carbohydrate intake appears to promote hepatic turnover to PA and subsequent urinary excretion [61].

Previous studies, although primarily epidemiological, have suggested that high-fat intake, particularly of SFA, may negatively influence one-carbon metabolites associated with CVD risk [19,20,23], whereas diets rich in complex carbohydrates tend to promote a more favorable profile [15,[17], [18], [19]]. In our study, however, where SFA intake was maintained at around 30 E% over a 1-y period, most risk-related metabolites—including tHcy—showed only minor within-group changes from baseline and no clear changes that would support any adverse effects. Interestingly, MMA displayed an opposite pattern to that reported in previous studies: levels increased in the A-HCLF group but decreased in the LCHF group, which suggests that diets higher in carbohydrates can contribute to raising MMA levels above thresholds associated with increased CVD risk [13,14]. Notably, MMA is sensitive to kidney function and can increase when renal function declines [63]. Potentially relevant to our findings, a study reported that lower-carbohydrate diets produced a greater increase in kidney function, measured by estimated glomerular filtration rate (eGFR), compared with diets higher in carbohydrate content and particularly high-carbohydrate, high-glycemic index diets [64]. In the current study, the main difference between the A-HCLF and C-HCLF diets was the degree of carbohydrate processing, with the A-HCLF diet consisting of a higher intake of more matrix-degraded carbohydrate sources, expected to have a higher glycemic index compared with the minimally processed carbohydrate sources in the C-HCLF. A carbohydrate processing-dependent impact on kidney function might therefore have contributed to the greater increase in MMA observed in the A-HCLF group compared with the C-HCLF and LCHF. Unfortunately, we did not measure eGFR in the current study, which would have provided better insight into this potential mechanism.

MMA is also known as a functional marker of vitamin B-12 status, as its accumulation typically results from impaired conversion of methylmalonyl-CoA to succinyl-CoA due to vitamin B-12 deficiency [65]. One possible contribution to the observed MMA changes could also be the slight reduction in vitamin B-12 intake in the A-HCLF group (−1.36 μg/d), compared with the increase in the LCHF group (+0.56 μg/d). For LCHF, the reduction in MMA further aligns with MMA reflecting changes in vitamin B-12 status. However, the C-HCLF group did not show the same increase in MMA as the A-HCLF group, despite showing the largest reduction in vitamin B-12 intake (−2.04 μg/d) and plasma cobalamin. Our findings therefore suggest an additional effect of carbohydrate quality on MMA levels that goes beyond reduced vitamin B-12 intake or status. Differences in carbohydrate quality may alter gut carbohydrate fermentation processes, thereby influencing the production of short-chain fatty acids such as propionic acid, a precursor to MMA [66,67]. Interestingly, addition of MMA to cultured hepatocytes has also been shown to decrease cholesterol synthesis while enhancing LDL receptor–mediated cholesterol uptake [68]. The increase in MMA observed in both HCLF groups might therefore potentially contribute to greater LDL clearance and attenuated hepatic cholesterol synthesis, and thereby potentially to relatively lower circulating cholesterol levels on such diets. Conversely, in the LCHF context in which circulating LDL cholesterol tends to increase, lower MMA might limit LDL recycling by the liver due to reduced LDL receptor activity. It remains speculative that reduced LDL cholesterol clearance by the liver increases LDL cholesterol on an SFA-rich LCHF diet via sterol-mediated downregulation of LDL receptors, as observed with high saturated fat intake in other contexts [69,70]. Direct evidence for such a mechanism during carbohydrate restriction is lacking, and a possible contributing role for MMA and vitamin B-12 in adapting cholesterol and lipid homeostasis in response to a saturated fat-rich LCHF diet warrants further investigation.

### Strengths and limitations

This study has both strengths and limitations. First, the presented analyses are secondary outcomes of the study and should therefore be considered exploratory. Furthermore, in addition to being isocaloric and isoproteinic, the study also aimed to match protein sources as closely as possible. However, given the substantial differences in carbohydrate amount between the diets, some variation in protein sources as well as intake of micronutrients, including B-vitamins, is inevitable. Moreover, missing plasma samples for some participants resulted in an incomplete dataset for the metabolite analyses. Also, although fasting plasma measurements provide a snapshot of the net circulating metabolite concentrations, they do not provide insight into tissue-specific metabolic changes. In addition, although samples were collected under fasting conditions, several of the included metabolites are known to fluctuate with time since the last meal, potentially introducing some bias [71]. However, given the magnitude and consistency of the observed between-group differences, it is unlikely that postprandial variation alone accounts for these findings. It is also important to note that several metabolites, such as MMA, PA, and tHcy, can be sensitive to kidney function [63,72]. As such, we cannot rule out that an improvement in kidney function may have contributed to the observed changes in the LCHF diet. Moreover, the current study included both males and females (20–56 y) of Caucasian origin with obesity, but our results may not be generalizable to other populations, such as older individuals, individuals without obesity, or those of non-Caucasian origin. Lastly, despite efforts to support adherence and reduce dropout rates, attrition was substantial. This loss of follow-up reduced the effective sample size at later visits and thereby reduced precision. Consequently, statistical power to detect clinically relevant differences was limited, particularly after 9 to 12 mo, and the statistically significant findings may be more prone to overestimating the underlying effect magnitude (type M error) [73]. Although the cLMM framework leverages all available repeated measurements and is more efficient than complete-case analyses under a missing-at-random assumption [39,74], it cannot fully address potential bias if dropout is differential between groups or related to unobserved outcomes. To assess the robustness to potential dropout-related bias, we performed IPW sensitivity analyses, which were highly consistent with the primary ITT estimates from the cLMMs, with only 1 metabolite showing a change in nominal significance. This concordance suggests that any bias introduced by differential dropout is likely to be limited, though inferences at later follow-up time points should still be interpreted with appropriate caution. The sensitivity analyses adjusting for diet adherence, however, had a greater impact on nominal significance patterns, indicating that variations in adherence to the diets contributed more meaningfully to shifts in statistical significance than dropout alone.

In conclusion, taken together, we found that a ketogenic low-carbohydrate diet high in total and saturated fat elicited significantly greater changes in plasma one-carbon metabolites and B-vitamer concentrations when compared with the more processed A-HCLF reference diet, whereas the minimally processed C-HCLF elicited smaller changes relative to the same comparator. As a notable example, our study indicates that not only vitamin B-12 status but diet quality, including macronutrient composition, can influence the CVD risk marker MMA. The more pronounced changes observed in the LCHF group may reflect physiological adaptations to carbohydrate restriction and increased fat intake. Lastly, our study shows that macronutrient composition and diet quality can have a marked impact on plasma levels of one-carbon and B-vitamer concentrations, independent of differences in dietary B-vitamin intake.

## Author contributions

The authors’ responsibilities were as follows – GAL, ON, GM, JD, SND: designed research; CH: conducted research; JL-B, MB: analyzed data; AM, JL-B, TB: curated data; MB, SND: wrote the paper and primary responsible for the final content; and all authors: read and approved the final manuscript.

## Data availability

Data described in the manuscript will be made available on request pending application and approval, with restrictions due to the sensitive nature of the data.

## Funding

This work was supported by Stiftelsen Dam, Oslo, Norway; Trond Mohn Research Foundation, Bergen, Norway; Kostfonden, Stockholm, Sweden.

## Declaration of Generative AI and AI-Assisted Technologies in the Writing Process

The authors declare that no generative AI or AI-assisted technologies were used in the writing of this manuscript.

## Conflict of interest

The authors report no conflicts of interest.
