# Supplementary material for: Marked Changes in One-Carbon Metabolism on a Low-Carbohydrate High-Fat Diet: A Randomized Controlled Trial (CARBFUNC)
Source: J Nutr. 2026 Jun 2;156(7):101625. doi: 10.1016/j.tjnut.2026.101625 (PMC13494189; doi:10.1016/j.tjnut.2026.101625)
Supplement: Supplementary file 1 [file mmc1.pdf]

Supplementary material

Marked changes in one-carbon metabolism on a low-carbohydrate high-fat diet: a randomized controlled trial (CARBFUNC)

Marianne Bråtveit[1,2], Johnny Laupsa–Borge[1,3], Tonje Bjørnstad[1,2], Cathrine Sommersten[1,4], Adrian McCann[3], Gülen Arslan Lied[5,6], Ottar Nygård[1,7], Gunnar Mellgren[1,2], Jutta Dierkes[5], Simon Nitter Dankel[1,2]

[1]Centre for Nutrition, Department of Clinical Science, University of Bergen, Bergen, Norway.

[2]Hormone Laboratory, Department of Medical Biochemistry and Pharmacology, Haukeland University Hospital, Bergen, Norway.

[3]Bevital AS, Bergen, Norway.

[4]Kunnskapskommunen Helse Omsorg Vest, Department of Ageing, Health and Care, Bergen, Norway.

[5]Centre for Nutrition, Department of Clinical Medicine, University of Bergen, Norway.

[6]Section of Gastroenterology, Department of Medicine, Haukeland University Hospital, Bergen, Norway.

[7]Department of Heart Disease, Haukeland University Hospital, Bergen, Norway.

Corresponding author: Simon N. Dankel, Department of Clinical Science, Jonas Lies veg 87, 5021 Bergen Norway, +47 943 08 637, simon.dankel@uib.no

Clinical Trial Registry number: NCT03401970.

Supplementary Tables:

Supplementary Table 1. Between-group difference q-values (to control for false discovery rate) from the primary intention-to-treat analysis. 3
Supplementary Table *2*. Within-group relative change scores from baseline for plasma one-carbon metabolites and B-vitamin status markers after 3, 6, 9, and 12 months. 5
Supplementary Table 3. Between-group differences in absolute change scores from baseline in dietary B-vitamin intake after 3, 6, 9, and 12 months[a] 9
Supplementary Table 4. Within-group absolute change scores from baseline in dietary B-vitamin intake after 3, 6, 9 and 12 months. 11
Supplementary Table 5. Comparison of primary ITT and diet adherence adjusted ITT analyses for between-group differences in change scores over time[a] 13
Supplementary Table 6. Comparison of primary and inverse probability weighted (IPW) analyses for nominal significance status in the plasma metabolites[a] 15
Supplementary Table 7. Comparison of primary and inverse probability weighted (IPW) analyses for nominal significance status in dietary B-vitamin intakes[a] 17

Supplementary Figures:

Supplementary Figure 1. Flow diagram of the study. 18
Supplementary Figure 2. Spearman correlation matrix of absolute changes in dietary B-vitamin intake against absolute changes in plasma B-vitamin status markers from baseline to 3 months in the A-HCLF group. 19
Supplementary Figure 3. Spearman correlation matrix of absolute changes in dietary B-vitamin intake against absolute changes in plasma B-vitamin status markers from baseline to 3 months in the C-HCLF group. 20
Supplementary Figure 4. Spearman correlation matrix of absolute changes in dietary B-vitamin intake against absolute changes in plasma B-vitamin status markers from baseline to 3 months in the LCHF group. 21

Supplementary Tables

Supplementary Table 1. Between-group difference q-values (to control for false discovery rate) from the primary intention-to-treat analysis.

Variable	3 months q-value	6 months q-value	9 months q-value	12 months q-value
**Methionine**				
LCHF	0.006	0.067	0.002	0.445
C-HCLF	0.501	0.949	0.428	0.946
**tHcy**				
LCHF	0.869	0.800	0.579	0.952
C-HCLF	0.999	0.979	0.821	0.946
**Cystathionine**				
LCHF	0.224	0.653	0.754	0.977
C-HCLF	0.782	0.463	0.967	0.955
**Cysteine**				
LCHF	0.069	0.275	0.676	0.815
C−HCLF	0.575	0.949	0.934	0.965
**α-hydroxybutyrate**				
LCHF	<0.001	0.002	0.004	0.769
C−HCLF	0.623	0.949	0.718	0.996
**Choline**				
LCHF	0.349	0.779	0.273	0.479
C−HCLF	0.619	0.949	0.967	0.834
**Betaine**				
LCHF	0.907	0.709	0.367	0.479
C−HCLF	0.575	0.949	0.967	0.834
**DMG**				
LCHF	0.742	0.800	0.686	0.726
C-HCLF	0.501	0.949	0.890	0.836
**Glycine**				
LCHF	0.772	0.810	0.640	0.790
C-HCLF	0.575	0.949	0.967	0.949
**Serine**				
LCHF	0.247	0.176	0.020	0.445
C-HCLF	0.575	0.949	0.821	0.836
**Sarcosine**				
LCHF	0.923	0.810	0.391	0.951
C-HCLF	0.684	0.949	0.967	0.946
**Riboflavin**				
LCHF	0.349	0.351	0.295	0.769
C-HCLF	0.790	0.975	0.807	0.946
**FMN**				
LCHF	0.932	0.800	0.349	0.994
C-HCLF	0.763	0.949	0.844	0.946
**NAM**				
LCHF	0.005	0.916	0.145	0.928
C-HCLF	0.134	0.949	0.572	0.962
**mNAM**				
LCHF	0.018	0.880	0.042	0.928
C-HCLF	0.154	0.949	0.063	0.946
**PLP**				
LCHF	0.344	0.458	0.308	0.999
C-HCLF	0.790	0.949	0.821	0.834
**PL**				
LCHF	0.018	0.176	0.075	0.999

C-HCLF	0.898	0.949	0.821	0.836
**PA**				
LCHF	<0.001	0.054	0.021	0.840
C-HCLF	0.864	0.949	0.967	0.834
**Folate, nmol/L**				
LCHF	0.687	0.548	0.400	0.769
C-HCLF	0.575	0.949	0.731	0.834
**Cobalamin, pmol/L**				
LCHF	0.685	0.779	0.228	0.977
C-HCLF	0.620	0.949	0.967	0.949
**MMA, umol/L**				
LCHF	<0.001	0.067	0.062	0.339
C-HCLF	0.501	0.949	0.430	0.996

[a] Values are q-values obtained using the Benjamini-Hochberg procedure for between-group differences in change from baseline from the primary intention-to-treat mixed model. The A-HCLF intervention was used as the comparator. *Abbreviations: A-HCLF, acellular high-carbohydrate low-fat diet; C-HCLF, cellular high-carbohydrate low-fat diet; DMG, dimethylglycine; FMN, flavin mononucleotide; LCHF, low-carbohydrate high-fat diet; MMA, methylmalonic acid; mNAM, 1-methylnicotinamide; NAM, nicotinamide; PA, 4-pyridoxic acid; PL, pyridoxal; PLP, pyridoxal 5'-phosphate; tHcy, total homocysteine.*

Supplementary Table 2. Within-group relative change scores from baseline for plasma one-carbon metabolites and B-vitamin status markers after 3, 6, 9, and 12 months.

	% change after 3 months[a]	P-value[a]	q-value[a]	% change after 6 months[b]	P-value[b]	q-value[b]	% change after 9 months[c]	P-value[c]	q-value[c]	% change after 12 months[d]	P-value[d]	q-value[d]
**Methionine, µmol/L**												
A-HCLF	1.44 (−2.82, 5.88)	0.513	0.710	3.18 (−3.60, 10.4)	0.365	0.508	4.90 (−0.20, 10.2)	0.060	0.161	−0.16 (−6.37, 6.47)	0.962	0.991
LCHF	−7.81 (−11.1, −4.45)	<0.001	<0.001	−9.12 (−14.0, −3.97)	0.001	0.003	−8.41 (−12.5, −4.17)	<0.001	0.001	−7.08 (−11.9, −2.02)	0.007	0.024
C-HCLF	−3.70 (−7.48, 0.24)	0.065	0.158	0.92 (−4.67, 6.84)	0.752	0.824	−1.74 (−6.16, 2.89)	0.453	0.604	1.67 (−3.57, 7.18)	0.539	0.704
**tHcy, µmol/L**												
A-HCLF	4.34 (−0.10, 8.98)	0.056	0.153	3.37 (−2.08, 9.12)	0.230	0.361	5.65 (−0.77, 12.5)	0.086	0.188	−0.98 (−6.39, 4.76)	0.732	0.848
LCHF	3.12 (−1.59, 8.05)	0.197	0.249	5.96 (−0.41, 12.7)	0.067	0.122	1.57 (−5.88, 9.61)	0.688	0.732	0.16 (−6.95, 7.82)	0.965	0.973
C-HCLF	4.28 (−0.60, 9.39)	0.086	0.178	3.49 (−2.41, 9.74)	0.251	0.381	2.76 (−4.23, 10.3)	0.447	0.602	1.65 (−4.68, 8.41)	0.616	0.760
**Cystathionine, µmol/L**												
A-HCLF	8.54 (−12.8, 35.1)	0.462	0.671	2.51 (−14.0, 22.2)	0.782	0.846	38.8 (9.19, 76.3)	0.008	0.036	11.2 (−11.7, 39.9)	0.365	0.573
LCHF	41.9 (16.5, 72.9)	0.001	0.001	20.9 (0.66, 45.2)	0.042	0.085	28.5 (0.20, 64.7)	0.048	0.091	13.2 (−10.8, 43.6)	0.305	0.425
C-HCLF	19.5 (−2.21, 46.1)	0.081	0.178	37.1 (19.4, 57.5)	<0.001	<0.001	40.7 (13.7, 74.0)	0.002	0.009	16.2 (−4.25, 41.0)	0.128	0.244
**Cysteine, µmol/L**												
A-HCLF	1.10 (−1.51, 3.78)	0.411	0.623	0.24 (−2.53, 3.09)	0.865	0.906	−0.89 (−4.10, 2.42)	0.593	0.739	−1.80 (−4.78, 1.27)	0.247	0.476
LCHF	−3.00 (−5.08, −0.88)	0.006	0.011	−3.48 (−5.64, −1.27)	0.002	0.007	−2.20 (−5.06, 0.73)	0.139	0.209	−3.17 (−5.60, −0.68)	0.013	0.039
C-HCLF	−0.98 (−3.36, 1.47)	0.430	0.562	−1.50 (−3.80, 0.85)	0.208	0.335	−1.46 (−4.42, 1.60)	0.345	0.506	−1.58 (−4.03, 0.93)	0.215	0.350
**α-hydroxybutyrate, µmol/L**												
A-HCLF	−1.56 (−11.9, 9.94)	0.780	0.876	−17.9 (−27.7, −6.74)	0.003	0.017	−16.2 (−26.0, −5.14)	0.005	0.029	−13.5 (−25.1, −0.14)	0.048	0.181
LCHF	41.3 (27.4, 56.6)	<0.001	<0.001	19.9 (6.24, 35.3)	0.003	0.009	16.6 (2.16, 33.1)	0.023	0.051	−5.47 (−17.9, 8.78)	0.431	0.534
C-HCLF	5.14 (−4.81, 16.1)	0.322	0.472	−8.30 (−17.0, 1.37)	0.090	0.182	−8.77 (−18.0, 1.53)	0.092	0.210	−13.4 (−22.4, −3.42)	0.010	0.037
**Choline, µmol/L**												
A-HCLF	−1.14 (−6.68, 4.72)	0.695	0.826	0.27 (−6.77, 7.83)	0.943	0.950	0.99 (−5.14, 7.52)	0.757	0.846	1.29 (−5.19, 8.21)	0.703	0.839
LCHF	−6.34 (−10.7, −1.71)	0.008	0.014	−3.84 (−9.37, 2.02)	0.194	0.285	−5.26 (−10.6, 0.38)	0.067	0.118	−5.10 (−10.4, 0.49)	0.073	0.144
C-HCLF	−4.95 (−9.95, 0.33)	0.066	0.158	−7.33 (−12.9, −1.44)	0.016	0.059	1.44 (−4.30, 7.52)	0.630	0.741	−5.78 (−11.1, −0.16)	0.044	0.119
**Betaine, µmol/L**												
A-HCLF	7.84 (1.10, 15.0)	0.022	0.078	11.4 (2.74, 20.9)	0.009	0.035	5.44 (−2.85, 14.4)	0.204	0.344	12.7 (2.64, 23.8)	0.012	0.117
LCHF	6.50 (0.93, 12.4)	0.022	0.035	5.40 (−1.36, 12.6)	0.120	0.190	13.2 (4.97, 22.1)	0.001	0.004	2.67 (−5.32, 11.3)	0.523	0.616
C-HCLF	1.63 (−4.32, 7.94)	0.599	0.706	2.23 (−4.62, 9.57)	0.532	0.644	4.71 (−2.97, 13.0)	0.235	0.415	1.72 (−6.06, 10.2)	0.673	0.802

	% change after 3 months[a]	P-value[a]	q-value[a]	% change after 6 months[b]	P-value[b]	q-value[b]	% change after 9 months[c]	P-value[c]	q-value[c]	% change after 12 months[d]	P-value[d]	q-value[d]
**DMG, µmol/L**												
A-HCLF	7.11 (−1.00, 15.9)	0.087	0.213	7.43 (−3.47, 19.6)	0.188	0.333	0.99 (−8.27, 11.2)	0.840	0.894	2.82 (−7.74, 14.6)	0.614	0.821
LCHF	3.94 (−2.68, 11.0)	0.249	0.297	2.80 (−5.81, 12.2)	0.535	0.648	4.83 (−4.01, 14.5)	0.293	0.394	−4.34 (−12.8, 4.98)	0.348	0.469
C-HCLF	−2.97 (−9.87, 4.46)	0.422	0.562	−2.33 (−10.9, 7.05)	0.614	0.711	−1.67 (−10.1, 7.56)	0.712	0.781	−5.41 (−13.8, 3.77)	0.238	0.379
**Glycine, µmol/L**												
A-HCLF	1.92 (−2.17, 6.18)	0.362	0.575	5.12 (−2.03, 12.8)	0.164	0.313	2.52 (−2.85, 8.20)	0.363	0.533	0.38 (−4.16, 5.14)	0.871	0.940
LCHF	3.33 (−0.12, 6.89)	0.059	0.083	7.85 (1.87, 14.2)	0.010	0.024	5.04 (0.12, 10.2)	0.045	0.089	2.67 (−1.23, 6.73)	0.182	0.302
C-HCLF	−1.56 (−5.25, 2.27)	0.418	0.562	1.76 (−4.12, 8.00)	0.565	0.666	1.89 (−3.05, 7.08)	0.460	0.607	1.45 (−2.53, 5.59)	0.479	0.652
**Serine, µmol/L**												
A-HCLF	2.80 (−1.12, 6.87)	0.164	0.332	2.69 (−2.26, 7.89)	0.291	0.422	0.063 (−3.94, 4.23)	0.976	0.983	−3.25 (−8.16, 1.93)	0.213	0.447
LCHF	7.40 (3.99, 10.9)	<0.001	<0.001	10.5 (6.20, 15.1)	<0.001	<0.001	8.74 (4.82, 12.8)	<0.001	<0.001	2.60 (−1.74, 7.12)	0.244	0.370
C-HCLF	−0.86 (−4.38, 2.80)	0.641	0.716	3.93 (−0.28, 8.31)	0.067	0.156	1.89 (−1.89, 5.82)	0.330	0.495	1.01 (−3.28, 5.49)	0.650	0.787
**Sarcosine, µmol/L**												
A-HCLF	4.48 (−3.62, 13.3)	0.286	0.508	5.40 (−4.62, 16.5)	0.301	0.427	6.70 (−0.72, 14.7)	0.077	0.183	6.64 (−3.45, 17.8)	0.204	0.436
LCHF	5.93 (−0.96, 13.3)	0.093	0.125	9.24 (0.76, 18.4)	0.032	0.068	13.0 (5.69, 20.8)	<0.001	0.001	4.52 (−3.71, 13.5)	0.290	0.411
C-HCLF	−0.025 (−7.29, 7.80)	0.995	0.995	3.26 (−5.05, 12.3)	0.452	0.563	7.30 (0.35, 14.7)	0.039	0.118	0.22 (−7.61, 8.71)	0.957	0.986
**Riboflavin, nmol/L**												
A-HCLF	−18.1 (−27.7, −7.19)	0.002	0.013	−4.67 (−19.5, 12.9)	0.578	0.688	−6.61 (−21.2, 10.7)	0.428	0.614	−19.0 (−32.9, −2.15)	0.029	0.153
LCHF	−27.1 (−34.3, −19.1)	<0.001	<0.001	−22.5 (−32.5, −11.1)	<0.001	0.001	−21.0 (−32.1, −8.04)	0.002	0.007	−27.7 (−38.6, −14.9)	<0.001	0.001
C-HCLF	−14.0 (−23.5, −3.38)	0.011	0.041	−3.41 (−16.4, 11.6)	0.637	0.720	−14.1 (−26.7, 0.57)	0.059	0.158	−13.2 (−26.5, 2.50)	0.095	0.205
**FMN, nmol/L**												
A-HCLF	0.54 (−8.62, 10.6)	0.912	0.955	14.1 (2.12, 27.6)	0.020	0.062	8.67 (−1.23, 19.6)	0.088	0.188	−7.28 (−20.6, 8.32)	0.340	0.556
LCHF	1.85 (−5.68, 9.99)	0.638	0.681	8.83 (−0.60, 19.2)	0.067	0.122	19.3 (7.58, 32.4)	0.001	0.003	−6.53 (−19.4, 8.39)	0.370	0.486
C-HCLF	−3.65 (−11.2, 4.51)	0.369	0.512	16.9 (9.85, 24.5)	<0.001	<0.001	12.1 (3.50, 21.5)	0.005	0.022	−2.68 (−16.3, 13.2)	0.724	0.845
**NAM, nmol/L**												
A-HCLF	14.0 (−2.42, 33.2)	0.098	0.236	1.18 (−20.9, 29.4)	0.925	0.946	−0.58 (−18.1, 20.7)	0.953	0.983	−15.8 (−32.7, 5.44)	0.134	0.327
LCHF	−19.7 (−29.1, −9.00)	0.001	0.002	−3.50 (−20.7, 17.5)	0.721	0.796	−23.7 (−36.1, −8.87)	0.003	0.008	−10.8 (−26.6, 8.30)	0.247	0.370
C-HCLF	−11.5 (−20.5, −1.40)	0.027	0.079	−17.3 (−30.6, −1.58)	0.033	0.101	−15.3 (−26.4, −2.52)	0.021	0.074	−17.3 (−28.7, −3.99)	0.013	0.046
**mNAM, nmol/L**												
A-HCLF	14.1 (−1.44, 32.2)	0.077	0.199	8.20 (−11.9, 32.8)	0.450	0.577	18.6 (−1.20, 42.4)	0.067	0.171	−11.2 (−28.7, 10.6)	0.287	0.525
LCHF	−14.1 (−24.0, −2.95)	0.015	0.025	2.94 (−12.9, 21.6)	0.733	0.799	−14.9 (−28.2, 0.68)	0.060	0.111	−6.01 (−22.2, 13.6)	0.520	0.616
C-HCLF	−11.5 (−22.8, 1.54)	0.081	0.178	−12.6 (−26.4, 3.91)	0.127	0.223	−21.2 (−33.5, −6.64)	0.006	0.024	−16.8 (−30.7, 0.044)	0.051	0.126

	% change after 3 months[a]	P-value[a]	q-value[a]	% change after 6 months[b]	P-value[b]	q-value[b]	% change after 9 months[c]	P-value[c]	q-value[c]	% change after 12 months[d]	P-value[d]	q-value[d]
**PLP, nmol/L**												
A-HCLF	20.6 (8.30, 34.4)	0.001	0.008	29.4 (9.10, 53.4)	0.003	0.018	24.1 (4.77, 46.9)	0.013	0.050	15.0 (0.70, 31.4)	0.039	0.161
LCHF	8.95 (−0.47, 19.3)	0.063	0.089	8.56 (−5.60, 24.8)	0.248	0.338	5.82 (−9.26, 23.4)	0.469	0.590	14.9 (2.34, 28.9)	0.019	0.052
C-HCLF	26.0 (13.9, 39.4)	<0.001	<0.001	20.6 (4.10, 39.8)	0.013	0.056	15.5 (−1.31, 35.1)	0.073	0.181	38.3 (23.4, 55.0)	<0.001	<0.001
**PL, nmol/L**												
A-HCLF	18.9 (7.12, 32.0)	0.001	0.010	30.9 (12.2, 52.7)	0.001	0.007	17.6 (2.81, 34.6)	0.018	0.064	3.06 (−10.1, 18.2)	0.666	0.821
LCHF	−2.42 (−10.6, 6.55)	0.584	0.633	3.52 (−8.80, 17.5)	0.592	0.697	−5.17 (−16.3, 7.38)	0.402	0.520	3.07 (−8.31, 15.9)	0.611	0.702
C-HCLF	21.3 (10.0, 33.8)	<0.001	0.001	18.2 (3.44, 35.0)	0.014	0.059	11.7 (−1.47, 26.6)	0.084	0.201	14.7 (1.98, 29.0)	0.022	0.074
**PA, nmol/L**												
A-HCLF	21.4 (9.06, 35.2)	<0.001	0.005	26.1 (7.57, 47.8)	0.004	0.022	15.6 (−0.57, 34.4)	0.059	0.161	10.0 (−6.52, 29.5)	0.249	0.476
LCHF	−12.4 (−20.0, −4.18)	0.004	0.008	−8.09 (−19.3, 4.73)	0.205	0.297	−14.5 (−25.6, −1.80)	0.027	0.058	2.94 (−10.4, 18.2)	0.680	0.761
C-HCLF	24.8 (12.8, 38.0)	<0.001	<0.001	14.0 (−0.67, 30.8)	0.062	0.152	14.7 (−0.33, 31.9)	0.056	0.158	27.7 (10.9, 47.0)	0.001	0.005
**Folate, nmol/L**												
A-HCLF	17.9 (5.01, 32.3)	0.005	0.028	26.0 (7.03, 48.4)	0.006	0.026	19.2 (−0.68, 42.9)	0.059	0.161	15.6 (−2.45, 36.9)	0.094	0.264
LCHF	24.4 (12.0, 38.2)	<0.001	<0.001	7.12 (−7.56, 24.1)	0.359	0.470	38.2 (15.5, 65.3)	<0.001	0.002	4.50 (−11.4, 23.2)	0.599	0.694
C-HCLF	5.82 (−7.83, 21.5)	0.421	0.562	−1.80 (−18.3, 18.0)	0.845	0.878	3.80 (−15.9, 28.1)	0.727	0.781	−5.89 (−22.8, 14.7)	0.546	0.706
**Cobalamin, pmol/L**												
A-HCLF	−4.46 (−11.0, 2.52)	0.204	0.384	−5.67 (−13.6, 2.97)	0.191	0.333	−7.43 (−15.2, 1.10)	0.086	0.188	1.92 (−8.07, 13.0)	0.718	0.839
LCHF	−1.06 (−6.69, 4.91)	0.721	0.755	−1.32 (−8.23, 6.12)	0.719	0.796	1.99 (−5.63, 10.2)	0.618	0.685	0.87 (−7.68, 10.2)	0.847	0.902
C-HCLF	−8.88 (−14.9, −2.48)	0.007	0.031	−11.9 (−18.6, −4.58)	0.002	0.011	−6.72 (−14.2, 1.44)	0.104	0.232	4.24 (−5.10, 14.5)	0.385	0.546
**MMA, μmol/L**												
A-HCLF	8.64 (3.86, 13.6)	<0.001	0.005	10.2 (4.51, 16.3)	<0.001	0.005	9.11 (2.81, 15.8)	0.004	0.027	7.51 (1.04, 14.4)	0.022	0.137
LCHF	−5.69 (−9.66, −1.53)	0.008	0.014	−0.68 (−5.60, 4.50)	0.792	0.850	−1.69 (−7.27, 4.22)	0.565	0.665	−2.11 (−8.21, 4.40)	0.515	0.616
C-HCLF	3.62 (0.039, 7.33)	0.048	0.131	3.42 (−0.89, 7.91)	0.121	0.216	1.30 (−3.83, 6.71)	0.625	0.741	7.48 (1.40, 13.9)	0.015	0.054

Fasting plasma levels of one-carbon metabolites and B-vitamers were analyzed with cLMMs. Values were transformed by the natural logarithm before the analyses. The relative change scores from baseline to follow-up are reported as percentages (95% CIs) calculated from cLMM estimates: $\% = (\exp^{\text{estimate}} - 1) \times 100$.

[a] Relative model-adjusted within-group mean change scores (95 % CIs) from baseline to 3 months, p-values and q-values (to control for false discovery rate using the Benjamini-Hochberg procedure) from the cLMMs. At 3 months, data from a total of 116 participants were available (A-HCLF, n = 32; C-HCLF, n = 37; LCHF, n = 47).

[b] Relative model-adjusted within-group mean change scores (95 % CIs) from baseline to 6 months, p-values and q-values (to control for false discovery rate using the Benjamini-Hochberg procedure) from the cLMMs. At 6 months, data from a total of 70 participants were available (A-HCLF, n = 17; C-HCLF, n = 26; LCHF, n = 27).

[c] Relative model-adjusted within-group mean change scores (95 % CIs) from baseline to 9 months, p-values and q-values (to control for false discovery rate using the Benjamini-Hochberg procedure) from the cLMMs. At 9 months, data from a total of 63 participants were available (A-HCLF, n = 19; C-HCLF, n = 22; LCHF, n = 22).

[d] Relative model-adjusted within-group mean change scores (95 % CIs) from baseline to 12 months, p-values and q-values (to control for false discovery rate using the Benjamini-Hochberg procedure) from the cLMMs. At 12 months, data from a total of 57 participants were available (A-HCLF, n = 14; C-HCLF, n = 22; LCHF, n = 21).

Abbreviations: A-HCLF, acellular high-carbohydrate low-fat diet; C-HCLF, cellular high-carbohydrate low-fat diet; DMG, dimethylglycine; FMN, flavin mononucleotide; LCHF, low-carbohydrate high-fat diet; MMA, methylmalonic acid; mNAM, 1-methylnicotinamide; NAM, nicotinamide; PA, 4-pyridoxic acid; PL, pyridoxal; PLP, pyridoxal 5'-phosphate; tHcy, total homocysteine.

Supplementary Table 3. Between-group differences in absolute change scores from baseline in dietary B-vitamin intake after 3, 6, 9, and 12 months[a]

Variable	Difference after 3 months vs. A-HCLF[b]	P-value[b]	q-value[b]	Difference after 6 months vs. A-HCLF[c]	P-value[c]	q-value[c]	Difference after 9 months vs. A-HCLF[d]	P-value[d]	q-value[d]	Difference after 12 months vs. A-HCLF[e]	P-value[e]	q-value[e]
**Vitamin B2, mg**												
LCHF	−0.21 (−0.31, −0.11)	<0.001	<0.001	−0.018 (−0.15, 0.12)	0.794	0.901	−0.062 (−0.24, 0.12)	0.497	0.522	−0.11 (−0.29, 0.081)	0.265	0.348
C-HCLF	−0.32 (−0.43, −0.22)	<0.001	<0.001	−0.19 (−0.33, −0.058)	0.005	0.022	−0.23 (−0.41, −0.050)	0.012	0.103	−0.27 (−0.45, −0.080)	0.005	0.076
**Vitamin B3, mg**												
LCHF	−2.30 (−3.88, −0.72)	0.005	0.006	−1.15 (−3.73, 1.43)	0.380	0.443	−2.46 (−5.88, 0.95)	0.156	0.212	−2.08 (−5.94, 1.77)	0.289	0.357
C-HCLF	−0.48 (−2.08, 1.13)	0.561	0.604	−0.55 (−3.15, 2.06)	0.679	0.792	−2.36 (−5.74, 1.03)	0.172	0.505	−2.42 (−6.26, 1.41)	0.214	0.361
**Niacin equivalents, mg**												
LCHF	−3.25 (−5.94, −0.57)	0.018	0.022	0.18 (−3.87, 4.24)	0.929	0.985	−3.02 (−9.03, 2.99)	0.323	0.388	0.51 (−5.81, 6.83)	0.874	0.874
C-HCLF	−0.32 (−3.04, 2.41)	0.818	0.838	−0.17 (−4.27, 3.92)	0.933	0.977	−3.29 (−9.24, 2.66)	0.278	0.556	−2.20 (−8.48, 4.09)	0.492	0.626
**Vitamin B6, mg**												
LCHF	−0.37 (−0.53, −0.22)	<0.001	<0.001	−0.17 (−0.44, 0.091)	0.196	0.257	−0.38 (−0.70, −0.061)	0.020	0.028	−0.41 (−0.71, −0.11)	0.007	0.015
C-HCLF	0.41 (0.25, 0.57)	<0.001	<0.001	0.42 (0.15, 0.68)	0.002	0.015	0.20 (−0.11, 0.52)	0.204	0.505	0.19 (−0.11, 0.49)	0.215	0.361
**Folate, µg**												
LCHF	−128 (−160, −96.2)	<0.001	<0.001	−81.0 (−129, −33.3)	0.001	0.001	−120 (−176, −64.2)	<0.001	<0.001	−129 (−196, −60.8)	<0.001	<0.001
C-HCLF	12.3 (−20.1, 44.7)	0.456	0.532	17.3 (−30.9, 65.4)	0.481	0.652	0.96 (−54.6, 56.5)	0.973	0.973	−25.0 (−92.6, 42.7)	0.469	0.615
**Vitamin B12, µg**												
LCHF	1.94 (1.17, 2.71)	<0.001	<0.001	2.57 (1.22, 3.93)	<0.001	<0.001	2.67 (1.29, 4.06)	<0.001	<0.001	2.31 (0.81, 3.81)	0.003	0.006
C-HCLF	−0.67 (−1.45, 0.11)	0.090	0.130	0.89 (−0.48, 2.26)	0.203	0.327	−0.44 (−1.80, 0.93)	0.531	0.673	−0.61 (−2.08, 0.86)	0.417	0.584

[a]Data were analysed with cLMMs (intention to treat). The A-HCLF dietary group was defined as the reference group in the between-group comparisons.
[b]Absolute model-adjusted between-group mean change score (95 % CIs) from baseline to 3 months, p-values and q-values (to control for false discovery rate using the Benjamini-Hochberg procedure) from the cLMMs. At 3 months, data on vitamin intake for a total of 163 participants were available (A-HCLF, n = 53; C-HCLF, n = 53; LCHF, n = 57).
[c]Absolute model-adjusted between-group mean change score (95 % CIs) from baseline to 6 months, p-values and q-values (to control for false discovery rate using the Benjamini-Hochberg procedure) from the cLMMs. At 6 months, data on vitamin intake for a total of 82 participants (A-HCLF, n = 23; C-HCLF, n = 29; LCHF, n = 30).
[d]Absolute model-adjusted between-group mean change score (95 % CIs) from baseline to 9 months, p-values and q-values (to control for false discovery rate using the Benjamini-Hochberg procedure) from the cLMMs. At 9 months, data on vitamin intake for a total of 63 participants were available (A-HCLF, n = 19; C-HCLF, n = 23; LCHF, n = 21).

[e]Absolute model-adjusted between-group mean change score (95 % CIs) from baseline to 12 months, p-values and q-values (to control for false discovery rate using the Benjamini-Hochberg procedure) from the cLMMs. At 12 months, data on vitamin intake for a total of 52 participants were available (A-HCLF, n = 12; C-HCLF, n = 21; LCHF, n = 19).

Abbreviations: A-HCLF, acellular high-carbohydrate low-fat diet; C-HCLF, cellular high-carbohydrate low-fat diet; cLMM, constrained linear mixed-effects model; LCHF, low-carbohydrate high-fat diet.

Supplementary Table 4. Within-group absolute change scores from baseline in dietary B-vitamin intake after 3, 6, 9 and 12 months.

Variable	Change after 3 months[a]	P-value[a]	q-value[a]	Change after 6 months[b]	P-value[b]	q-value[b]	Change after 9 months[c]	P-value[c]	q-value[c]	Change after 12 months[d]	P-value[d]	q-value[d]
**Vitamin B2, mg**												
A−HCLF	−0.10 (−0.32, 0.13)	0.399	0.409	−0.28 (−0.52, −0.046)	0.019	0.035	−0.26 (−0.50, −0.015)	0.038	0.088	−0.27 (−0.52, −0.015)	0.038	0.096
LCHF	−0.31 (−0.53, −0.081)	0.008	0.011	−0.30 (−0.53, −0.067)	0.012	0.017	−0.32 (−0.56, −0.080)	0.009	0.014	−0.37 (−0.62, −0.13)	0.003	0.006
C−HCLF	−0.42 (−0.65, −0.19)	<0.001	0.001	−0.48 (−0.71, −0.24)	<0.001	<0.001	−0.49 (−0.73, −0.25)	<0.001	<0.001	−0.53 (−0.78, −0.29)	<0.001	<0.001
**Vitamin B3, mg**												
A−HCLF	1.78 (0.16, 3.41)	0.031	0.040	1.27 (−0.93, 3.47)	0.257	0.338	2.19 (−0.55, 4.94)	0.117	0.179	2.50 (−0.72, 5.73)	0.128	0.256
LCHF	−0.51 (−2.11, 1.09)	0.530	0.543	0.12 (−1.90, 2.13)	0.910	0.962	−0.27 (−2.91, 2.37)	0.841	0.841	0.42 (−2.46, 3.30)	0.773	0.792
C−HCLF	1.31 (−0.32, 2.94)	0.114	0.133	0.72 (−1.33, 2.77)	0.489	0.501	−0.16 (−2.77, 2.44)	0.903	0.903	0.08 (−2.77, 2.93)	0.956	0.956
**Niacin equivalents, mg**												
A−HCLF	4.46 (2.03, 6.88)	<0.001	0.001	1.73 (−1.58, 5.03)	0.305	0.366	4.14 (−0.48, 8.76)	0.079	0.144	3.47 (−1.67, 8.62)	0.185	0.310
LCHF	1.20 (−1.17, 3.57)	0.319	0.344	1.91 (−1.11, 4.93)	0.214	0.257	1.12 (−3.30, 5.53)	0.619	0.650	3.98 (−0.52, 8.49)	0.083	0.112
C−HCLF	4.14 (1.71, 6.56)	0.001	0.001	1.55 (−1.53, 4.63)	0.322	0.356	0.85 (−3.48, 5.19)	0.699	0.716	1.28 (−3.18, 5.73)	0.573	0.602
**Vitamin B6, mg**												
A−HCLF	0.32 (0.03, 0.60)	0.029	0.038	0.17 (−0.14, 0.48)	0.278	0.344	0.34 (0.015, 0.67)	0.041	0.090	0.31 (−0.007, 0.64)	0.055	0.121
LCHF	−0.06 (−0.34, 0.22)	0.678	0.678	−0.00 (−0.30, 0.30)	0.984	0.984	−0.04 (−0.36, 0.28)	0.818	0.838	−0.10 (−0.40, 0.20)	0.520	0.546
C−HCLF	0.73 (0.44, 1.01)	<0.001	<0.001	0.59 (0.29, 0.89)	<0.001	<0.001	0.55 (0.23, 0.86)	0.001	0.002	0.50 (0.21, 0.80)	0.001	0.003
**Folate, µg**												
A−HCLF	165 (133, 196)	<0.001	<0.001	131 (88.1, 175)	<0.001	<0.001	145 (99.5, 191)	<0.001	<0.001	148 (91.9, 203)	<0.001	<0.001
LCHF	36.5 (5.57, 67.3)	0.021	0.027	50.4 (9.81, 91.1)	0.015	0.021	25.3 (−18.6, 69.3)	0.258	0.292	19.0 (−31.5, 69.6)	0.460	0.495
C−HCLF	177 (145, 208)	<0.001	<0.001	149 (107, 190)	<0.001	<0.001	146 (102, 190)	<0.001	<0.001	123 (72.4, 173)	<0.001	<0.001
**Vitamin B12, µg**												
A−HCLF	−1.37 (−2.44, −0.30)	0.013	0.019	−2.19 (−3.54, −0.83)	0.002	0.005	−1.53 (−2.87, −0.19)	0.025	0.062	−1.30 (−2.76, 0.15)	0.079	0.165
LCHF	0.57 (−0.49, 1.64)	0.289	0.328	0.39 (−0.89, 1.67)	0.553	0.628	1.14 (−0.16, 2.45)	0.086	0.112	1.00 (−0.28, 2.29)	0.127	0.156
C−HCLF	−2.04 (−3.11, −0.97)	<0.001	<0.001	−1.30 (−2.59, −0.004)	0.049	0.077	−1.97 (−3.25, −0.68)	0.003	0.008	−1.91 (−3.17, −0.65)	0.003	0.007

Data were analysed with cLMMs.

[a]Absolute model-adjusted within-group mean change scores (95 % CIs) from baseline to 3 months, p-values and q-values (to control for false discovery rate using the Benjamini-Hochberg procedure) from the cLMMs. At 3 months, data on vitamin intake for a total of 163 participants were available (A-HCLF, n = 53; C-HCLF, n = 53; LCHF, n = 57).
[b]Absolute model-adjusted within-group mean change scores (95 % CIs) from baseline to 6 months, p-values and q-values (to control for false discovery rate using the Benjamini-Hochberg procedure) from the cLMMs. At 6 months, data on vitamin intake for a total of 82 participants (A-HCLF, n = 23; C-HCLF, n = 29; LCHF, n = 30).
[c]Absolute model-adjusted within-group mean change scores (95 % CIs) from baseline to 9 months, p-values and q-values (to control for false discovery rate using the Benjamini-Hochberg procedure) from the cLMMs. At 9 months, data on vitamin intake for a total of 63 participants were available (A-HCLF, n = 19; C-HCLF, n = 23; LCHF, n = 21).
[d]Absolute model-adjusted within-group mean change scores (95 % CIs) from baseline to 12 months, p-values and q-values (to control for false discovery rate using the Benjamini-Hochberg procedure) from the cLMMs. At 12 months, data on vitamin intake for a total of 52 participants were available (A-HCLF, n = 12; C-HCLF, n = 21; LCHF, n = 19).
Abbreviations: A-HCLF, acellular high-carbohydrate low-fat diet; C-HCLF, cellular high-carbohydrate low-fat diet; cLMM, constrained linear mixed-effects model; LCHF, low-carbohydrate high-fat diet.

Supplementary Table 5. Comparison of primary ITT and diet adherence adjusted ITT analyses for between-group differences in change scores over time[a]

Variable	Change scores 3M		Change scores 6M		Change scores 9M		Change scores 12M	
	Primary ITT	Diet adherence adjusted ITT	Primary ITT	Diet adherence adjusted ITT	Primary ITT	Diet adherence adjusted ITT	Primary ITT	Diet adherence adjusted ITT
**Methionine, µmol/L**								
LCHF vs. A-HCLF	-9.12 (-13.9, -4.06)	-8.47 (-14.3, -2.25)	-11.9 (-19.2, -3.99)	-12.3 (-21.1, -2.66)	-12.7 (-18.3, -6.70)	-13.7 (-20.8, -6.02)	-6.94 (-14.3, 1.07)	-5.14 (-14.6, 5.39)
C-HCLF vs. A-HCLF	-5.06 (-10.3, 0.52)	-4.47 (-9.81, 1.18)	-2.19 (-10.4, 6.75)	-2.24 (-11.3, 7.70)	**-6.33 (-12.4, 0.15)**	**-8.25 (-14.6, -1.41)**	1.83 (-6.22, 10.6)	7.61 (-2.10, 18.3)
**tHcy, µmol/L**								
LCHF vs. A-HCLF	-1.17 (-7.16, 5.21)	-0.90 (-8.04, 6.79)	2.52 (-5.43, 11.1)	4.22 (-5.62, 15.1)	-3.86 (-12.8, 6.00)	-3.18 (-15.4, 10.9)	1.15 (-7.70, 10.8)	-2.49 (-11.5, 7.44)
C-HCLF vs. A-HCLF	-0.059 (-6.21, 6.49)	0.017 (-6.35, 6.82)	0.12 (-7.41, 8.27)	1.64 (-7.01, 11.1)	-2.74 (-11.4, 6.78)	-3.45 (-11.7, 5.60)	2.65 (-5.66, 11.7)	-0.64 (-9.39, 8.97)
**Cystathionine, µmol/L**								
LCHF vs. A-HCLF	**30.7 (-2.49, 75.3)**	**52.5 (9.77, 112)**	17.9 (-8.30, 51.7)	30.2 (-5.96, 80.2)	-7.42 (-34.2, 30.2)	7.40 (-29.7, 64.1)	1.83 (-26.6, 41.2)	18.8 (-21.4, 79.5)
C-HCLF vs. A-HCLF	10.1 (-18.1, 48.0)	4.61 (-22.4, 41.0)	**33.8 (7.10, 67.1)**	**25.1 (-2.55, 60.7)**	1.38 (-26.2, 39.2)	-1.74 (-30.7, 39.4)	4.52 (-22.4, 40.7)	-7.57 (-33.7, 28.9)
**Cysteine, µmol/L**								
LCHF vs. A-HCLF	**-4.06 (-7.21, -0.80)**	**-3.46 (-7.20, 0.41)**	**-3.71 (-7.08, -0.22)**	**-3.51 (-7.55, 0.71)**	-1.33 (-5.55, 3.08)	-3.32 (-7.91, 1.50)	-1.40 (-5.20, 2.56)	-3.55 (-7.91, 1.01)
C-HCLF vs. A-HCLF	-2.05 (-5.44, 1.46)	-2.20 (-5.71, 1.44)	-1.74 (-5.24, 1.89)	-3.15 (-6.76, 0.60)	-0.57 (-4.88, 3.94)	-2.52 (-6.56, 1.70)	0.22 (-3.63, 4.23)	-0.46 (-4.42, 3.67)
**α-hydroxybutyrate, µmol/L**								
LCHF vs. A-HCLF	43.5 (24.6, 65.3)	61.4 (36.9, 90.3)	46.0 (23.2, 73.1)	68.2 (37.4, 106)	39.2 (16.7, 66.0)	67.2 (35.3, 107)	**9.29 (-10.4, 33.3)**	**27.9 (1.82, 60.6)**
C-HCLF vs. A-HCLF	6.80 (-7.07, 22.8)	1.98 (-11.5, 17.5)	11.7 (-4.55, 30.7)	8.33 (-8.53, 28.3)	8.86 (-7.12, 27.6)	7.11 (-9.21, 26.4)	0.10 (-16.3, 19.7)	-0.093 (-17.0, 20.2)
**Choline, µmol/L**								
LCHF vs. A-HCLF	-5.25 (-12.0, 1.97)	-6.52 (-14.3, 1.97)	-4.10 (-12.5, 5.16)	-6.04 (-15.8, 4.90)	**-6.19 (-13.7, 2.01)**	**-10.7 (-19.9, -0.41)**	-6.31 (-13.9, 1.95)	-5.90 (-16.0, 5.47)
C-HCLF vs. A-HCLF	-3.85 (-11.0, 3.89)	-3.53 (-10.8, 4.32)	-7.57 (-15.8, 1.51)	-7.70 (-16.4, 1.86)	0.44 (-7.65, 9.25)	0.69 (-8.17, 10.4)	-6.98 (-14.5, 1.26)	-3.76 (-13.1, 6.52)
**Betaine, µmol/L**								
LCHF vs. A-HCLF	-1.24 (-8.98, 7.16)	-4.17 (-13.2, 5.80)	-5.41 (-14.6, 4.78)	-8.84 (-19.5, 3.23)	7.37 (-3.77, 19.8)	0.85 (-12.1, 15.7)	-8.91 (-19.4, 2.90)	-7.03 (-20.0, 8.02)
C-HCLF vs. A-HCLF	-5.76 (-13.5, 2.71)	-4.58 (-12.6, 4.15)	-8.26 (-17.3, 1.83)	-5.96 (-15.7, 4.92)	-0.69 (-11.0, 10.9)	-1.84 (-12.8, 10.5)	-9.75 (-20.0, 1.85)	-5.59 (-17.6, 8.18)
**DMG, µmol/L**								
LCHF vs. A-HCLF	-2.96 (-12.2, 7.22)	-7.64 (-18.2, 4.27)	-4.31 (-16.5, 9.68)	-11.5 (-24.7, 4.06)	3.80 (-8.57, 17.8)	-4.27 (-19.1, 13.3)	-6.96 (-19.2, 7.09)	-11.8 (-26.9, 6.45)
C-HCLF vs. A-HCLF	-9.41 (-18.5, 0.64)	-8.52 (-17.8, 1.79)	-9.08 (-20.9, 4.50)	-7.64 (-20.1, 6.75)	-2.64 (-14.3, 10.7)	-1.62 (-14.8, 13.6)	-8.00 (-20.1, 5.87)	-6.09 (-20.8, 11.4)
**Glycine, µmol/L**								
LCHF vs. A-HCLF	1.38 (-3.81, 6.84)	-0.94 (-6.89, 5.38)	2.60 (-6.22, 12.3)	0.13 (-9.85, 11.2)	2.46 (-4.61, 10.0)	-1.76 (-9.93, 7.15)	2.28 (-3.61, 8.54)	0.16 (-7.32, 8.23)
C-HCLF vs. A-HCLF	-3.42 (-8.61, 2.08)	-2.59 (-7.88, 3.00)	-3.20 (-11.7, 6.08)	-2.48 (-11.7, 7.64)	-0.62 (-7.58, 6.85)	-1.74 (-9.04, 6.16)	1.06 (-4.84, 7.33)	3.44 (-3.29, 10.6)
**Serine, µmol/L**								
LCHF vs. A-HCLF	4.48 (-0.59, 9.80)	4.57 (-1.25, 10.7)	7.64 (1.11, 14.6)	9.15 (1.78, 17.1)	**8.67 (2.94, 14.7)**	**5.99 (-0.50, 12.9)**	**6.04 (-0.80, 13.4)**	**9.92 (1.63, 18.9)**
C-HCLF vs. A-HCLF	-3.55 (-8.48, 1.64)	-3.71 (-8.67, 1.51)	1.21 (-5.02, 7.84)	1.48 (-4.78, 8.15)	1.83 (-3.61, 7.58)	-1.09 (-6.20, 4.30)	**4.40 (-2.35, 11.6)**	**9.44 (2.02, 17.4)**
**Sarcosine, µmol/L**								
LCHF vs. A-HCLF	1.39 (-8.44, 12.3)	-2.21 (-13.3, 10.3)	3.64 (-8.69, 17.6)	-2.02 (-15.4, 13.4)	5.88 (-3.64, 16.3)	0.74 (-10.9, 13.9)	-1.98 (-13.7, 11.3)	-3.82 (-17.5, 12.2)

C-HCLF vs. A-HCLF	-4.32 (-14.1, 6.54)	-3.58 (-13.8, 7.80)	-2.03 (-13.9, 11.4)	-1.60 (-13.8, 12.4)	0.56 (-8.50, 10.5)	1.42 (-8.07, 11.9)	-6.02 (-17.2, 6.64)	-8.29 (-20.1, 5.27)
**Riboflavin, nmol/L**								
LCHF vs. A-HCLF	**-11.0 (-24.1, 4.32)**	**-22.9 (-36.0, -7.17)**	**-18.7 (-34.6, 0.91)**	**-30.9 (-46.2, -11.2)**	**-15.4 (-32.6, 6.13)**	**-31.1 (-47.5, -9.66)**	-10.8 (-30.4, 14.4)	-24.6 (-43.9, 1.26)
C-HCLF vs. A-HCLF	4.91 (-11.3, 24.1)	5.77 (-10.4, 24.9)	1.33 (-18.8, 26.4)	0.47 (-19.9, 26.0)	-8.06 (-27.0, 15.8)	-9.57 (-28.8, 14.9)	7.12 (-16.6, 37.6)	9.51 (-16.5, 43.5)
**FMN, nmol/L**								
LCHF vs. A-HCLF	1.31 (-10.3, 14.4)	0.95 (-11.6, 15.3)	-4.65 (-17.3, 9.89)	-0.32 (-15.3, 17.4)	9.81 (-4.48, 26.2)	12.5 (-4.29, 32.3)	0.81 (-18.6, 24.9)	5.68 (-15.6, 32.4)
C-HCLF vs. A-HCLF	-4.17 (-15.4, 8.59)	-4.31 (-15.6, 8.48)	2.45 (-9.76, 16.3)	4.08 (-8.36, 18.2)	3.19 (-8.84, 16.8)	-5.66 (-18.2, 8.82)	4.96 (-15.5, 30.4)	7.32 (-13.3, 32.8)
**NAM, nmol/L**								
LCHF vs. A-HCLF	-29.6 (-41.9, -14.6)	-21.2 (-37.7, -0.27)	-4.63 (-30.0, 29.9)	-0.52 (-31.0, 43.4)	**-23.3 (-40.6, -0.87)**	**-16.1 (-40.2, 17.8)**	5.86 (-20.9, 41.6)	26.9 (-15.1, 89.6)
C-HCLF vs. A-HCLF	-22.4 (-35.4, -6.70)	-23.7 (-36.9, -7.78)	-18.3 (-39.3, 9.95)	-23.2 (-44.2, 5.93)	-14.8 (-32.5, 7.56)	-12.1 (-32.6, 14.5)	-1.81 (-24.6, 27.8)	2.25 (-27.0, 43.3)
**mNAM, nmol/L**								
LCHF vs. A-HCLF	**-24.7 (-37.6, -9.28)**	**-8.99 (-27.6, 14.4)**	-4.86 (-26.6, 23.4)	19.3 (-12.4, 62.6)	**-28.3 (-43.6, -8.78)**	**-16.6 (-37.7, 11.7)**	**5.86 (-20.5, 41.0)**	**48.7 (5.06, 111)**
C-HCLF vs. A-HCLF	-22.4 (-36.3, -5.54)	-26.0 (-39.6, -9.43)	-19.2 (-37.9, 5.21)	-20.1 (-39.5, 5.54)	-33.6 (-47.8, -15.5)	-31.3 (-46.2, -12.4)	-6.24 (-29.3, 24.4)	-4.44 (-30.0, 30.5)
**PLP, nmol/L**								
LCHF vs. A-HCLF	-9.69 (-21.1, 3.35)	4.06 (-11.3, 22.1)	-16.1 (-32.6, 4.45)	1.19 (-21.8, 30.9)	-14.7 (-31.8, 6.59)	9.68 (-14.4, 40.6)	**-0.15 (-15.7, 18.3)**	**33.7 (8.73, 64.4)**
C-HCLF vs. A-HCLF	4.47 (-9.37, 20.4)	1.09 (-12.2, 16.4)	-6.76 (-25.5, 16.6)	-7.95 (-27.5, 16.9)	-6.94 (-25.7, 16.6)	-4.11 (-22.7, 19.0)	20.2 (1.59, 42.3)	27.9 (7.16, 52.6)
**PL, nmol/L**								
LCHF vs. A-HCLF	**-17.9 (-27.9, -6.65)**	**-12.9 (-25.0, 1.25)**	**-20.9 (-35.1, -3.63)**	**-15.9 (-33.2, 5.97)**	**-19.4 (-32.3, -3.99)**	**-8.65 (-25.0, 11.2)**	0.013 (-16.1, 19.2)	7.14 (-13.4, 32.5)
C-HCLF vs. A-HCLF	2.05 (-10.9, 16.9)	-2.14 (-14.4, 11.9)	-9.71 (-26.2, 10.5)	-13.4 (-30.1, 7.24)	-5.03 (-20.3, 13.2)	-2.48 (-17.3, 14.9)	11.3 (-6.66, 32.8)	5.55 (-12.9, 27.9)
**PA, nmol/L**								
LCHF vs. A-HCLF	-27.9 (-37.0, -17.4)	-20.5 (-32.2, -6.88)	**-27.1 (-40.6, -10.6)**	**-18.0 (-35.4, 4.16)**	**-26.0 (-39.4, -9.77)**	**-8.40 (-26.5, 14.2)**	-6.45 (-24.3, 15.5)	1.73 (-21.5, 31.8)
C-HCLF vs. A-HCLF	2.74 (-10.9, 18.5)	-3.97 (-16.4, 10.4)	-9.60 (-26.6, 11.3)	-16.7 (-33.1, 3.72)	-0.79 (-18.8, 21.2)	-0.17 (-17.0, 20.1)	16.0 (-6.22, 43.5)	-0.47 (-21.8, 26.6)
**Folate, nmol/L**								
LCHF vs. A-HCLF	**5.58 (-9.23, 22.8)**	**21.8 (0.33, 47.8)**	-15.0 (-31.7, 5.82)	0.52 (-23.2, 31.6)	**16.0 (-9.89, 49.2)**	**40.7 (4.60, 89.2)**	-9.57 (-28.4, 14.1)	11.0 (-16.3, 47.3)
C-HCLF vs. A-HCLF	-10.2 (-24.7, 7.00)	-13.8 (-28.0, 3.25)	-22.1 (-39.0, -0.51)	-24.5 (-42.0, -1.86)	-12.9 (-33.8, 14.6)	-12.8 (-34.1, 15.3)	-18.6 (-37.0, 5.22)	-7.69 (-30.2, 22.1)
**Cobalamin, pmol/L**								
LCHF vs. A-HCLF	3.56 (-5.39, 13.3)	3.73 (-6.81, 15.5)	4.61 (-6.48, 17.0)	5.62 (-7.34, 20.4)	10.2 (-1.75, 23.6)	7.67 (-5.80, 23.1)	-1.02 (-13.5, 13.2)	-7.33 (-21.1, 8.90)
C-HCLF vs. A-HCLF	-4.63 (-13.4, 5.04)	-5.96 (-15.0, 4.07)	-6.59 (-16.9, 4.97)	-8.42 (-18.9, 3.47)	0.78 (-10.5, 13.5)	-3.06 (-14.1, 9.39)	2.29 (-10.9, 17.4)	-3.69 (-17.4, 12.3)
**MMA, umol/L**								
LCHF vs. A-HCLF	-13.2 (-18.4, -7.68)	-12.7 (-18.9, -6.07)	-9.91 (-16.2, -3.14)	-9.86 (-17.9, -1.06)	-9.90 (-17.0, -2.22)	-10.3 (-19.4, -0.24)	-8.95 (-16.6, -0.57)	-12.3 (-21.6, -1.90)
C-HCLF vs. A-HCLF	-4.62 (-9.90, 0.96)	-4.04 (-9.76, 2.04)	-6.19 (-12.3, 0.37)	-4.11 (-11.2, 3.57)	-7.16 (-14.1, 0.37)	-7.09 (-14.9, 1.41)	-0.031 (-8.11, 8.76)	-0.45 (-9.42, 9.42)

[a] Relative between-group differences (95% CIs) from baseline for all metabolites at 3, 6, 9, and 12 months, comparing the primary intention-to-treat (ITT) analysis with the dietary-adherence adjusted ITT analysis. Values were natural log-transformed and analyzed with constrained linear mixed-effects models adjusted for baseline values, age, and sex. The A-HCLF intervention was used as the comparator. The diet adherence adjusted models were additionally adjusted for self-reported dietary adherence and deviations between planned and reported intake of energy and macronutrients expressed as energy percentage. Bold values indicate time points where nominal significance status differed between the primary ITT and diet adherence adjusted ITT.

Abbreviations: A-HCLF, acellular high-carbohydrate low-fat diet; C-HCLF, cellular high-carbohydrate low-fat diet; DMG, dimethylglycine; FMN, flavin mononucleotide; LCHF, low-carbohydrate high-fat diet; MMA, methylmalonic acid; mNAM, 1-methylnicotinamide; NAM, nicotinamide; PA, 4-pyridoxic acid; PL, pyridoxal; PLP, pyridoxal 5'-phosphate; tHcy, total homocysteine

Supplementary Table 6. Comparison of primary and inverse probability weighted (IPW) analyses for nominal significance status in the plasma metabolites[a]

Variable	3 months		6 months		9 months		12 months	
	Primary ITT	IPW	Primary ITT	IPW	Primary ITT	IPW	Primary ITT	IPW
**Methionine, µmol/L**								
LCHF vs. A-HCLF	0.001	0.001	0.004	0.004	0.000	0.000	0.088	0.086
C-HCLF vs. A-HCLF	0.074	0.073	0.618	0.620	0.055	0.055	0.666	0.663
**tHcy, µmol/L**								
LCHF vs. A-HCLF	0.711	0.711	0.545	0.545	0.428	0.428	0.806	0.806
C-HCLF vs. A-HCLF	0.985	0.985	0.976	0.976	0.559	0.559	0.542	0.542
**Cystathionine, µmol/L**								
LCHF vs. A-HCLF	0.073	0.081	0.198	0.285	0.657	0.662	0.913	0.786
C-HCLF vs. A-HCLF	0.522	0.562	**0.011**	**0.053**	0.932	0.928	0.770	0.695
**Cysteine, µmol/L**								
LCHF vs. A-HCLF	0.015	0.015	0.038	0.038	0.548	0.613	0.482	0.394
C-HCLF vs. A-HCLF	0.247	0.254	0.341	0.339	0.799	0.814	0.911	0.996
**α-hydroxybutyrate, µmol/L**								
LCHF vs. A-HCLF	0.000	0.000	0.000	0.000	0.000	0.000	0.379	0.424
C-HCLF vs. A-HCLF	0.353	0.402	0.167	0.186	0.294	0.299	0.991	0.944
**Choline, µmol/L**								
LCHF vs. A-HCLF	0.149	0.149	0.373	0.372	0.134	0.134	0.130	0.131
C-HCLF vs. A-HCLF	0.319	0.320	0.099	0.100	0.918	0.917	0.095	0.098
**Betaine, µmol/L**								
LCHF vs. A-HCLF	0.763	0.758	0.286	0.279	0.203	0.213	0.133	0.109
C-HCLF vs. A-HCLF	0.176	0.176	0.105	0.104	0.902	0.887	0.096	0.085
**DMG, µmol/L**								
LCHF vs. A-HCLF	0.554	0.558	0.526	0.536	0.564	0.565	0.313	0.299
C-HCLF vs. A-HCLF	0.065	0.066	0.179	0.180	0.681	0.683	0.243	0.236
**Glycine, µmol/L**								
LCHF vs. A-HCLF	0.608	0.608	0.575	0.573	0.504	0.502	0.455	0.475
C-HCLF vs. A-HCLF	0.218	0.217	0.485	0.486	0.865	0.875	0.729	0.747
**Serine, µmol/L**								
LCHF vs. A-HCLF	0.084	0.084	0.021	0.021	0.003	0.002	0.085	0.100
C-HCLF vs. A-HCLF	0.175	0.175	0.710	0.707	0.516	0.492	0.206	0.236
**Sarcosine, µmol/L**								
LCHF vs. A-HCLF	0.791	0.788	0.579	0.586	0.234	0.240	0.757	0.741
C-HCLF vs. A-HCLF	0.420	0.420	0.754	0.750	0.907	0.913	0.335	0.329
**Riboflavin, nmol/L**								
LCHF vs. A-HCLF	0.150	0.150	0.060	0.060	0.148	0.148	0.368	0.368
C-HCLF vs. A-HCLF	0.575	0.573	0.907	0.907	0.475	0.479	0.589	0.591
**FMN, nmol/L**								
LCHF vs. A-HCLF	0.833	0.787	0.510	0.418	0.187	0.222	0.941	0.968
C-HCLF vs. A-HCLF	0.503	0.588	0.708	0.696	0.618	0.552	0.661	0.667
**NAM, nmol/L**								
LCHF vs. A-HCLF	0.000	0.001	0.763	0.807	0.043	0.018	0.700	0.615
C-HCLF vs. A-HCLF	0.007	0.017	0.181	0.192	0.177	0.143	0.892	0.915
**mNAM, nmol/L**								
LCHF vs. A-HCLF	0.003	0.003	0.706	0.708	0.007	0.007	0.696	0.710
C-HCLF vs. A-HCLF	0.012	0.012	0.113	0.113	0.001	0.001	0.655	0.638
**PLP, nmol/L**								
LCHF vs. A-HCLF	0.138	0.138	0.116	0.116	0.161	0.161	0.986	0.973
C-HCLF vs. A-HCLF	0.545	0.546	0.539	0.537	0.530	0.527	0.032	0.035
**PL, nmol/L**								
LCHF vs. A-HCLF	0.003	0.003	0.020	0.020	0.016	0.016	0.999	0.995

C-HCLF vs. A-HCLF	0.768	0.768	0.321	0.321	0.563	0.563	0.232	0.230
**PA, nmol/L**								
LCHF vs. A-HCLF	0.000	0.000	0.002	0.002	0.003	0.005	0.535	0.486
C-HCLF vs. A-HCLF	0.709	0.759	0.341	0.382	0.938	0.843	0.171	0.266
**Folate, nmol/L**								
LCHF vs. A-HCLF	0.480	0.541	0.145	0.164	0.249	0.326	0.396	0.289
C-HCLF vs. A-HCLF	0.228	0.195	0.045	0.044	0.323	0.204	0.116	0.075
**Cobalamin, pmol/L**								
LCHF vs. A-HCLF	0.447	0.447	0.429	0.429	0.097	0.097	0.880	0.880
C-HCLF vs. A-HCLF	0.335	0.335	0.251	0.251	0.898	0.898	0.748	0.748
**MMA, umol/L**								
LCHF vs. A-HCLF	0.000	0.000	0.005	0.010	0.013	0.017	0.037	0.040
C-HCLF vs. A-HCLF	0.103	0.213	0.064	0.136	0.062	0.088	0.994	0.968

[a] Values are nominal p-values for between-group differences in change from baseline from the primary intention-to-treat mixed model and the inverse probability weighted (IPW) sensitivity analysis. The A-HCLF intervention was used as the comparator. Nominal significance was defined as $p < 0.05$. Bold values indicate time points where nominal significance status differed between the primary intention-to-treat and IPW analyses.

Abbreviations: A-HCLF, acellular high-carbohydrate low-fat diet; C-HCLF, cellular high-carbohydrate low-fat diet; DMG, dimethylglycine; FMN, flavin mononucleotide; LCHF, low-carbohydrate high-fat diet; MMA, methylmalonic acid; mNAM, 1-methylnicotinamide; NAM, nicotinamide; PA, 4-pyridoxic acid; PL, pyridoxal; PLP, pyridoxal 5'-phosphate; tHcy, total homocysteine.

Supplementary Table 7. Comparison of primary and inverse probability weighted (IPW) analyses for nominal significance status in dietary B-vitamin intakes[a]

	3 months		6 months		9 months		12 months	
**Variable**	**Primary ITT**	**IPW**	**Primary ITT**	**IPW**	**Primary ITT**	**IPW**	**Primary ITT**	**IPW**
**Vitamin B2, mg**								
LCHF	<0.001	<0.001	0.794	0.775	0.497	0.488	0.265	0.263
C-HCLF	<0.001	<0.001	0.005	0.005	0.012	0.011	0.005	0.005
**Vitamin B3, mg**								
LCHF	0.005	0.005	0.380	0.381	0.156	0.155	0.289	0.290
C-HCLF	0.561	0.561	0.679	0.680	0.172	0.172	0.214	0.219
**Niacin equivalents, mg**								
LCHF	0.018	0.017	0.929	0.933	0.323	0.319	0.874	0.896
C-HCLF	0.818	0.816	0.933	0.935	0.278	0.278	0.492	0.488
**Vitamin B6, mg**								
LCHF	<0.001	<0.001	0.196	0.197	0.020	0.020	0.007	0.008
C-HCLF	<0.001	<0.001	0.002	0.002	0.204	0.202	0.215	0.217
**Folate, µg**								
LCHF	<0.001	<0.001	0.001	0.001	<0.001	<0.001	<0.001	<0.001
C-HCLF	0.456	0.456	0.481	0.483	0.973	0.973	0.469	0.509
**Vitamin B12, µg**								
LCHF	<0.001	<0.001	<0.001	<0.001	<0.001	<0.001	0.003	0.004
C-HCLF	0.090	0.089	0.203	0.200	0.531	0.529	0.417	0.414

[a] Values are nominal p-values for between-group differences in change from baseline from the primary intention-to-treat mixed model and the inverse probability weighted (IPW) sensitivity analysis. The A-HCLF intervention was used as the comparator. Nominal significance was defined as $p < 0.05$.

Abbreviations: A-HCLF, acellular high-carbohydrate low-fat diet; C-HCLF, cellular high-carbohydrate low-fat diet; LCHF, low-carbohydrate high-fat diet.

Supplementary Figures

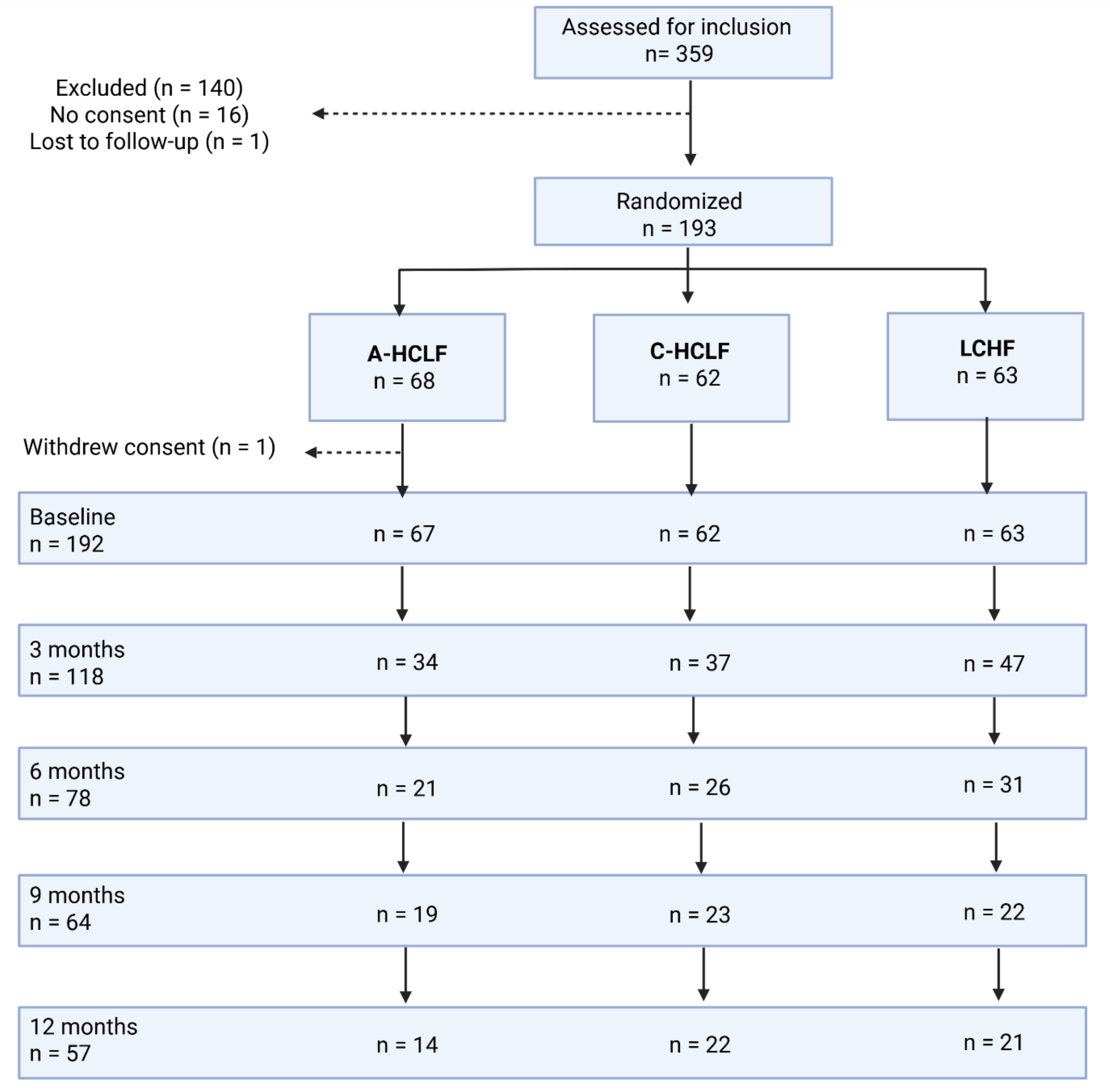

Supplementary Figure 1. Flow diagram of the study.
Abbreviations: A-HCLF, acellular high-carbohydrate low-fat diet; C-HCLF, cellular high-carbohydrate low-fat diet; LCHF, low-carbohydrate high-fat diet. Created in BioRender. Bråtveit, M. (2026) https://BioRender.com/ naeza16

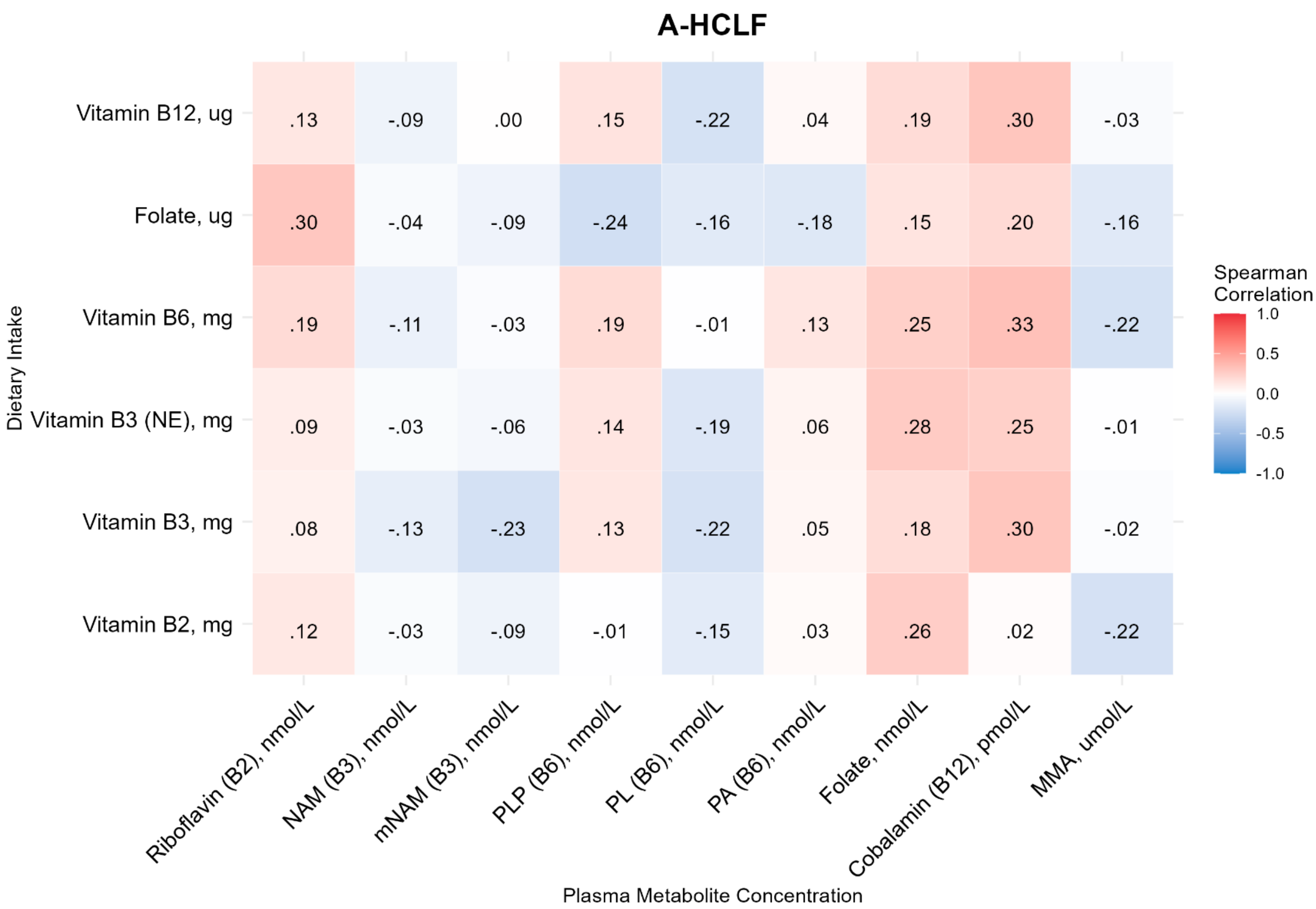

Supplementary Figure 2. Spearman correlation matrix of absolute changes in dietary B-vitamin intake against absolute changes in plasma B-vitamin status markers from baseline to 3 months in the A-HCLF group. Negative correlations are marked with a leading minus sign and colored blue while positive correlations are marked in red without a minus sign. A spearman correlation coefficient close to -1 indicates a strong negative correlation, while a spearman correlation coefficient close to +1 indicates a strong positive correlation. *Abbreviations: A-HCLF, acellular high-carbohydrate low-fat diet; MMA, methylmalonic acid; mNAM, 1-methylnicotinamide; NAM, nicotinamide; NE, niacin equivalents; PA, 4-pyridoxic acid; PL, pyridoxal; PLP, pyridoxal 5'-phosphate.*

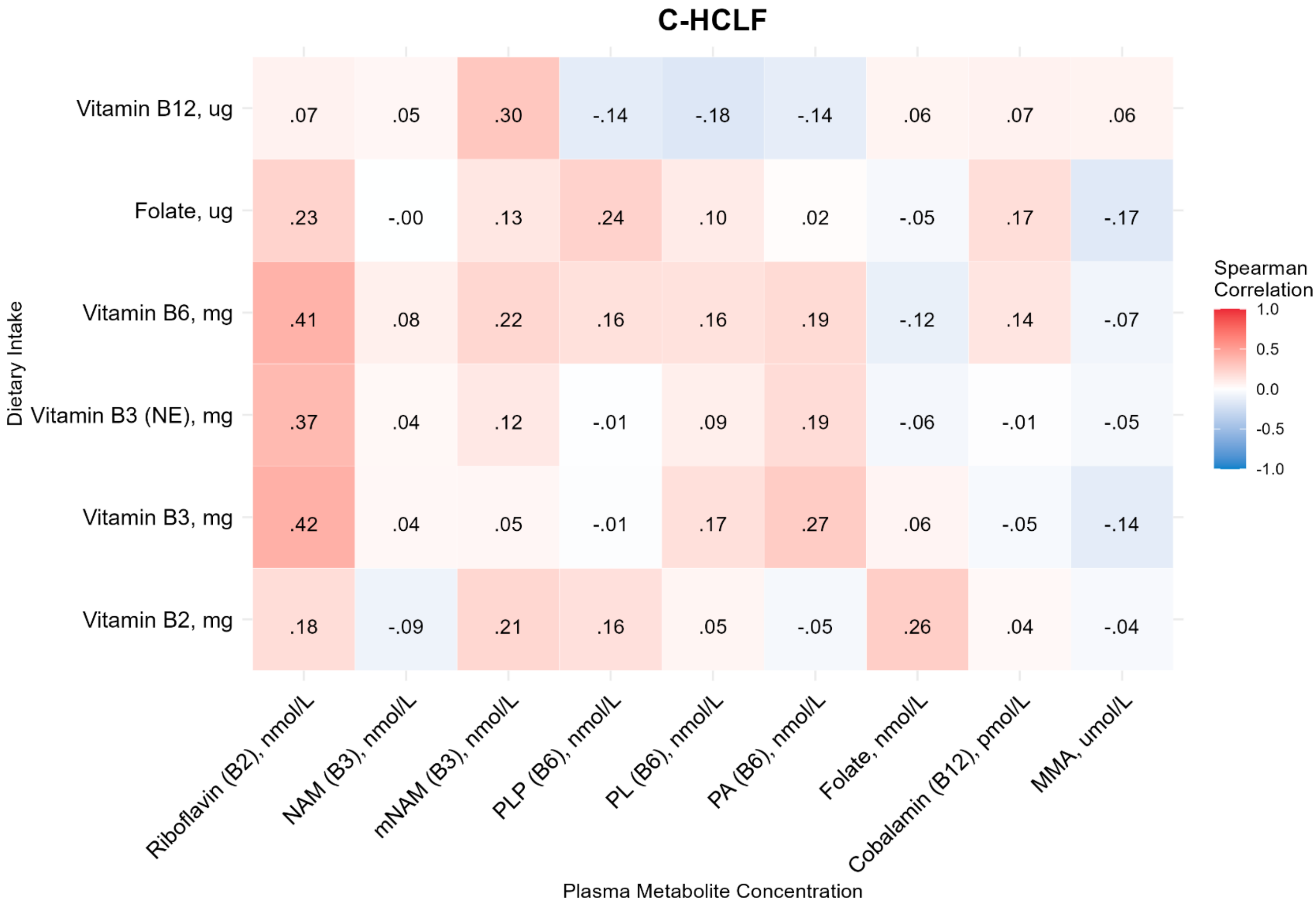

Supplementary Figure 3. Spearman correlation matrix of absolute changes in dietary B-vitamin intake against absolute changes in plasma B-vitamin status markers from baseline to 3 months in the C-HCLF group. Negative correlations are marked with a leading minus sign and colored blue while positive correlations are marked in red without a minus sign. A spearman correlation coefficient close to -1 indicates a strong negative correlation, while a spearman correlation coefficient close to +1 indicates a strong positive correlation. *Abbreviations: C-HCLF, cellular high-carbohydrate diet, MMA, methylmalonic acid; mNAM, 1-methylnicotinamide; NAM, nicotinamide; NE, niacin equivalents; PA, 4-pyridoxic acid; PL, pyridoxal; PLP, pyridoxal 5'-phosphate.*

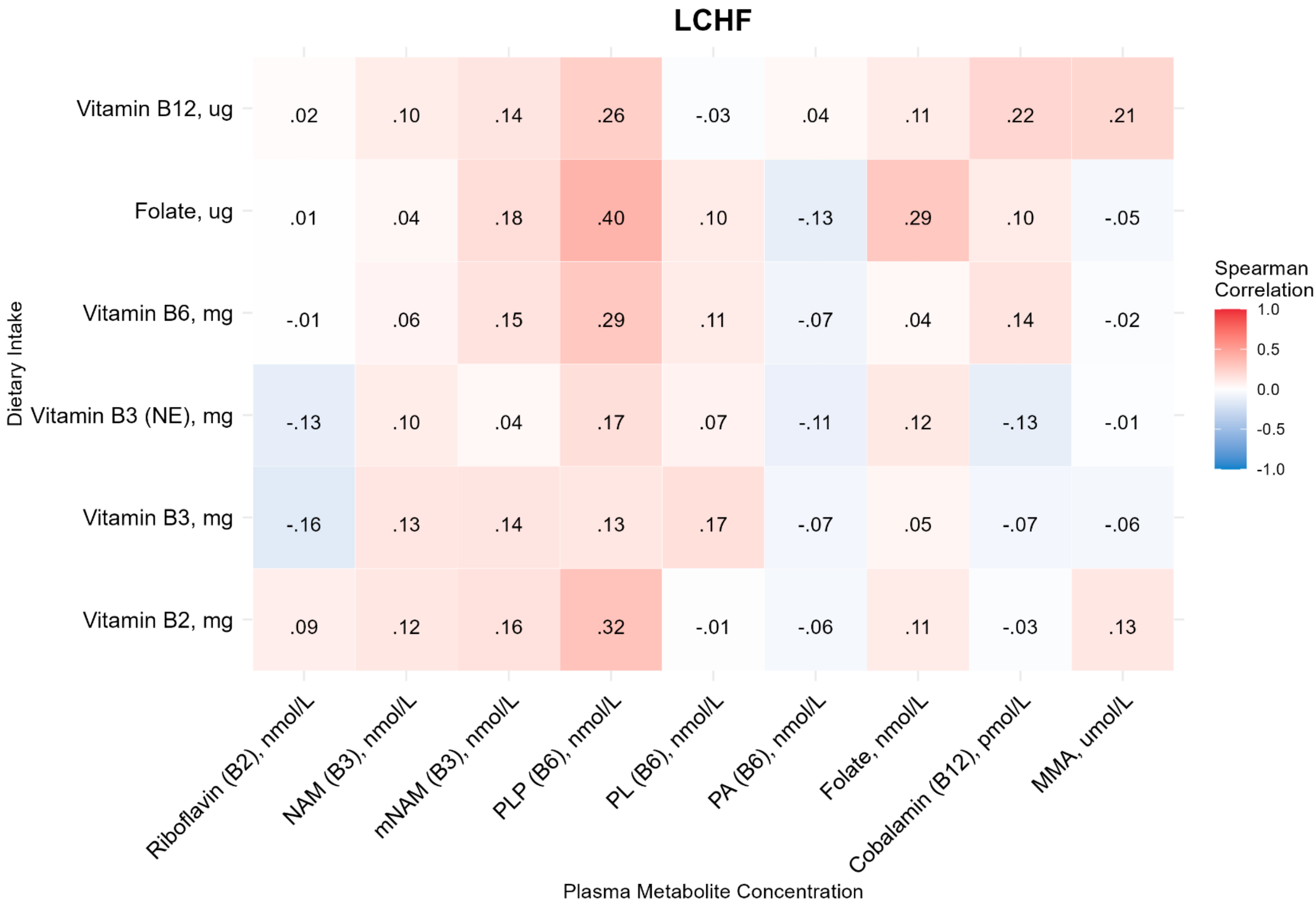

Supplementary Figure 4. Spearman correlation matrix of absolute changes in dietary B-vitamin intake against absolute changes in plasma B-vitamin status markers from baseline to 3 months in the LCHF group. Negative correlations are marked with a leading minus sign and colored blue while positive correlations are marked in red without a minus sign. A spearman correlation coefficient close to -1 indicates a strong negative correlation, while a spearman correlation coefficient close to +1 indicates a strong positive correlation. *Abbreviations: LCHF, low-carbohydrate high-fat diet; MMA, methylmalonic acid; mNAM, 1-methylnicotinamide; NAM, nicotinamide; NE, niacin equivalents; PA, 4-pyridoxic acid; PL, pyridoxal; PLP, pyridoxal 5'-phosphate.*
